# Pesticide‐Free Rice Paddies Promote Diverse and Distinct Aquatic Invertebrate Communities in Cool‐Temperate Agroecosystems

**DOI:** 10.1002/ece3.74287

**Published:** 2026-09-18

**Authors:** Thea Bulas, Benedikt R. Schmidt, Christoph Vorburger, Roel D'Haese, Yvonne Fabian

**Affiliations:** ^1^ Agricultural Landscapes and Biodiversity, Department of Agroecology and Environment Agroscope Zürich Switzerland; ^2^ D‐USYS, Department of Environmental Systems Science ETH Zürich Zürich Switzerland; ^3^ Department of Evolutionary Biology and Environmental Studies University of Zurich Zurich Switzerland; ^4^ Info Fauna Karch Neuchâtel Switzerland; ^5^ Department of Aquatic Ecology Eawag, Swiss Federal Institute of Aquatic Science and Technology Dübendorf Switzerland

**Keywords:** agroecology, aquatic biodiversity, land‐sharing, macroinvertebrate communities, rice paddy

## Abstract

Freshwater wetlands are rapidly declining due to the expansion of agriculture and urbanisation, resulting in significant biodiversity loss. In the Swiss lowlands, 90% of wetlands have been lost. Researchers and farmers are therefore jointly exploring the pesticide‐free cultivation of paddy rice as a land‐sharing approach that increases habitat availability for aquatic organisms. We studied the role of rice paddies for aquatic biodiversity by comparing key environmental variables and macroinvertebrate communities with those in nearby natural wetlands over a 2‐year period. Rice paddies may provide a more uniform aquatic environment than natural wetlands. Accordingly, wetlands harboured a higher taxon richness at the regional scale, but at the local scale, macroinvertebrate density and richness were higher in rice paddies (1212 [95% CI: 714–2058] vs. 448 [263–761] ind./m^2^ and 19.36 [17.36–21.59] vs. 13.39 [11.90–15.08] taxa/site, respectively). In contrast, Shannon diversity was higher in wetlands than in rice paddies (1.69 [1.51–1.87] vs. 1.46 [1.27–1.64]). Six rice paddies were constructed with a deeper side ditch, which may provide a distinct habitat element promoting additional taxa, but its overall effect on rice paddy communities was limited. These results highlight the ecological value of rice paddies in providing large areas of temporary habitat for freshwater species that can complement but not replace natural wetlands in Swiss agricultural landscapes. While they show some parallels to studies from warmer rice‐growing regions, our findings mainly apply to cold‐temperate climate and pesticide‐free cultivation, stressing the importance of effective management practices to maximise the ecological benefits of rice paddies.

## Graphical abstract

Freshwater wetlands are disappearing across agricultural landscapes, prompting interest in pesticide‐free rice paddies as multifunctional habitats. Across 2 years, rice paddies supported higher local macroinvertebrate density and richness than nearby natural wetlands, although wetlands maintained greater regional diversity and more heterogeneous environmental conditions. Taxa significantly linked to rice paddies were the Gastropod *Physa* sp. and the dragonfly *Orthetrum cancellatum*, with natural wetlands: *Sphaerium* sp., *Asellus aquaticus*, *Caenis* sp., Notonectoidea, *Plae minutissima* and *Micronecta* sp. These findings show that rice paddies can enhance habitat availability for freshwater taxa but remain complementary rather than substitutive to natural wetland conservation in intensively used landscapes.

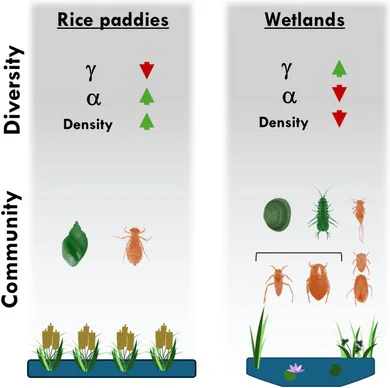

## 1 Introduction

Freshwater ecosystems are among the most threatened ecosystems globally. Biodiversity is declining at a faster rate in freshwater than in terrestrial or marine ecosystems (IPBES 2019). In the densely populated lowlands of Switzerland, only 10% of wetlands remain (Gimmi et al. 2011). This habitat loss is the leading driver of freshwater species decline, highlighting the need for wetland restoration and the creation of new wetlands (Davidson 2014; Fluet‐Chouinard et al. 2023; Verhoeven 2014; Verhoeven and Setter 2010), which demonstrably benefit amphibians and other aquatic taxa (Katayama et al. 2019, 2023; Moor et al. 2022; Thiere et al. 2009; Fahy et al. 2025).

In Switzerland, new opportunities for restoration arise from the ageing drainage infrastructure. Much of it was built around the mid‐20th century and becomes increasingly costly to maintain (Béguin and Smola 2010; Koch and Prasuhn 2020). These costs could be avoided if fields were managed in a way that does not require drainage. At the same time, rising temperatures make the Swiss climate increasingly suitable for rice production. Paddy rice cultivation thus presents an opportunity to combine agricultural production with wetland restoration on poorly drained soils. Additionally, strict regulations on agrochemicals (VBP 2023; WPO 1998) prohibit the use of pesticides in paddy rice, making Swiss rice production uniquely compatible with biodiversity conservation in a land‐sharing approach (Grass et al. 2019, 2021; Katayama et al. 2019, 2023; Kremen 2015). Since the first successful trials in 2017 (Jacot et al. 2018), biodiversity has become a primary objective of paddy rice cultivation, but the ecological benefits and limitations of these systems, especially compared to natural wetlands, remain insufficiently quantified (Echeverría‐Progulakis et al. 2026; Pérez‐Méndez et al. 2025).

Rice fields in Mediterranean, Asian or South American regions often support communities dominated by disturbance‐tolerant taxa. Their community structure is strongly shaped by management practices such as water regime or pesticide use (Bo et al. 2025; Echeverría‐Progulakis et al. 2026; Giuliano and Bogliani 2019; Nakanishi et al. 2025; Leitão et al. 2007; Lupi et al. 2013, 2014; Rosecchi et al. 2006; Suhling et al. 2000; Tawa et al. 2024; Watanabe et al. 2025). Studies from Portugal, Italy, and France indicate that field margins and habitat heterogeneity can promote additional taxa, but overall richness is often limited by short hydroperiods and uniform conditions (Bo et al. 2025; Leitão et al. 2007; Lupi et al. 2013, 2014; Echeverría‐Progulakis et al. 2026; Rosecchi et al. 2006). In Switzerland, paddy rice cultivation is recent, it takes place under a cool‐temperate climate in a landscape where wetlands are scarce, using low‐intensity practices without pesticides. This unique context allows Swiss paddies to serve as a biodiversity‐friendly model of rice cultivation. Our study provides the first systematic comparison of their aquatic macroinvertebrate communities with natural wetlands, offering new insights into land‐sharing conservation in agricultural landscapes.

**FIGURE 1:**
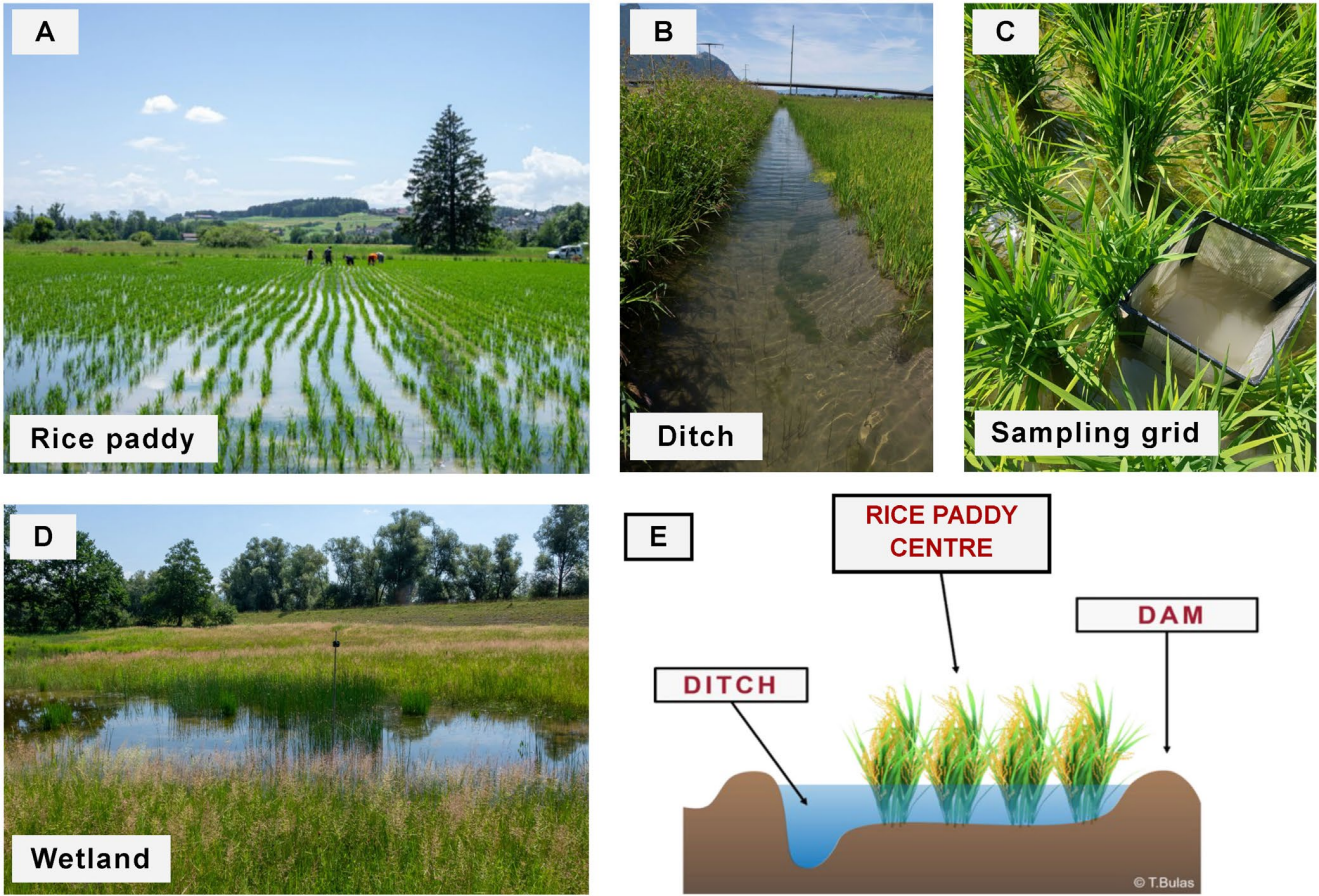
(A) Rice paddy during manual weeding (end of June 2022). (B) Ditch structure integrated within a rice paddy (mid‐June 2022). (C) Sampling grid used for collecting macroinvertebrates positioned between rice plants. (D) Wetland (June 2022, early season). (E) Schematic illustration of a rice paddy with an integrated ditch.

Rice paddies function as temporary wetlands (Odum 1997; Wellborn et al. 1996), favouring species with rapid development, drought‐resistant life stages, or colonisation abilities typical of temporary pond species (Bulas et al. 2025; Choe et al. 2016; Kidera et al. 2018; Leitão et al. 2007; Lupi et al. 2013, 2014; Natuhara 2013; Rosecchi et al. 2006; Schnapauff et al. 2000; Wiggins et al. 1980). The short hydroperiod of Swiss rice paddies with only one crop per year (Figure 1A) also prevents fish establishment, benefiting species sensitive to fish predation (e.g., certain amphibian tadpoles and dragonfly nymphs). Some species, such as dragonflies (Monnerat et al. 2021), may benefit from the nutrient input provided by fertilisers in rice crops, which can increase developmental rates and final adult size (Bulas et al. 2025). A subset of aquatic species (Bo et al. 2025), particularly those with long development times, may require additional features to complete their life‐cycles, such as adjacent ditches (Figure 1B,E). Ditches are channel‐like depressions along the side of rice paddies that are connected to the paddies and stay flooded for longer time (Natuhara 2013).

Although paddy rice can provide habitat for many species (Bo et al. 2025; Bambaradeniya et al. 2004; Bambaradeniya and Amerasinghe 2003; Katayama et al. 2019, 2023; Kidera et al. 2018; Leitão et al. 2007; Natuhara 2013; Rosecchi et al. 2006; Schnapauff et al. 2000; Yoon 2009), its effects on aquatic biodiversity in temperate regions and the effectiveness of management elements such as ditches remain unclear. Macroinvertebrates are widely recognised as sensitive indicators of aquatic ecosystem condition and biodiversity, as their community composition responds to changes in water quality, habitat structure, and management practices (Echeverría‐Progulakis et al. 2026; Pérez‐Méndez et al. 2025).

To explore the potential benefits of rice paddies for aquatic biodiversity in Switzerland, we studied 11 paddies and compared them with nearby wetlands (Figure 1D), measuring key environmental variables and sampling their macroinvertebrate communities over a two‐year period. Our study focused on two main comparisons. First, we compared rice paddies with nearby natural wetlands. Specifically, we studied (i) if and how environmental conditions differed between rice paddies and wetlands, (ii) how macroinvertebrate diversity in these two habitat types varied at local (alpha) and regional (gamma) scales, and (iii) which taxa were associated with either habitat type, thereby shaping community composition. Second, we compared rice paddies with and without ditches. Specifically, we investigated (i) whether the presence of ditches in paddies affected environmental conditions compared to paddies without ditches; (ii) how the presence of ditches influenced macroinvertebrate diversity in rice paddies; and (iii) whether the ditch habitat harboured particular taxa that were less common within the paddies and in paddies without ditches.

Drawing on ecological theory linking hydroperiod and habitat heterogeneity to aquatic community assembly (Wellborn et al. 1996; Wiggins et al. 1980), we formulated two main hypotheses. First, we hypothesised that rice paddies and wetlands would differ in their environmental conditions and macroinvertebrate community composition. Specifically, we expected rice paddies to support lower regional diversity and a distinct assemblage of taxa because they represent relatively homogenous temporary habitats compared with the broader range of environmental conditions present across wetlands. Second, we hypothesised that paddies containing ditches would support distinct macroinvertebrate communities compared to paddies without ditches because ditches provide additional, qualitatively different habitat with prolonged inundation relative to paddy centres. Consequently, we expected ditches to support additional taxa and contribute to diversity within the rice‐paddy system.

## 2 Methods

### 2.1 Study Area

This study was conducted in the Swiss lowlands north of the Alps, involving 11 rice paddies and nearby wetlands. We refer to such pairs as sites. The distance between the rice paddy and the wetland ranged from 150 to 1500 m (Table S1).

The annual average temperatures in the Swiss lowlands range from 8°C to 12°C (MeteoSwiss, n.d.). Precipitation varies from 1000 to 1500 mm per year, with more rainfall occurring in summer than in winter (MeteoSwiss, n.d.). The sizes of paddies ranged from 0.4 to 1.5 ha (Table S1) and those of wetlands from 0.0115 to 1.7 ha. Nine sites contained a single rice paddy, and two sites had multiple paddies. At the multi‐paddy sites, we sampled both paddies in Mühlau and three out of the six paddies in La Sauge (Table S1).

The rice paddies were flooded around the time of planting (with the rice variety *Loto*) in May. Before flooding, the paddies may be levelled and puddled to break down soil aggregates, improving water retention and ensuring an even water depth across the paddies. The water management is consistent with Mediterranean rice cultivation practices described by Kraehmer et al. (2017). The paddies were continuously flooded from mid‐May (transplantation) until about a month before the harvest in September. Throughout this period, water levels were maintained at around 10 cm. Between the end of July and mid‐August, the water supply was stopped to allow the fields to dry, making it easier for harvesters to harvest the crop. There was no mid‐season drainage or intermittent irrigation used during the growing season. While the fields may naturally refill for brief periods in autumn and winter due to rainfall or snowmelt, this happens outside the cropping season. Paddies were fertilised with organic (Biorga Quick, Cow manure, slurry) or mineral (Urea, Hauert Geistlich N12, EuroChem Entec26, Landor Nitrotec26) nitrous fertilisers (Widmer et al. 2025; Bulas et al. 2025), applied before flooding and transplantation, at quantities of up to 105 kg N/ha per season (Sinaj et al. 2017). Additional top‐dressing was applied later if rice showed nutrient deficiencies, especially during active tillering and panicle initiation. No pesticides were used because Swiss law restricts their use in open water bodies (VBP 2023; WPO 1998).

The 11 wetlands (Table S1) included in this study consist of a range of aquatic habitats located near the rice paddies. Temporary wetlands occasionally dried during the study period, whereas permanent wetlands remained inundated throughout the year. Nine wetlands were isolated water bodies located close to rivers, while two were directly connected to the Aare River and the Fosse des Talons, respectively. Fish were present in four wetlands and absent from the remaining seven.

Of the 11 paddies, six had ditches running along one edge of the paddy (Table S1), typically adjacent to the levee on the irrigation inlet side. In three fields, ditches ran along two or three edges of the paddy. All ditches were directly connected to the main paddy (Figure 1E) and flooded simultaneously. After the main paddy was drained, ditches generally remained flooded until harvest or occasionally even longer, depending on rainfall and local drainage conditions. We categorised ditch presence as a binary variable (present/absent) for analysis.

### 2.2 Macroinvertebrate Sampling

We sampled macroinvertebrates in 11 rice paddies and 11 wetlands during the crop growing period mid‐ to late‐season in 2022 and 2023, with two sampling occasions each year (July and August). Macroinvertebrates were collected by exhaustive net sweeping (0.5 mm mesh) within a 25 cm × 25 cm polycarbonate frame (Figure 1C), including the top sediment layer, until no further individuals were caught. The 0.5 mm mesh was chosen as it retains most macroinvertebrate taxa while minimising clogging by fine debris. The number of subsamples was roughly proportional to water surface area, with 4–25 sub‐samples taken per site (0.0115–1.7 ha). Subsamples were distributed throughout each microhabitat to obtain representative site‐level coverage. Within rice paddies, samples were collected from both central and peripheral areas following a spatially balanced field traversal and pooled into a composite sample for subsequent analyses. Ditches were sampled along their length and the subsamples pooled separately. Densities (individuals per m^2^) were calculated as the total number of individuals divided by the total sampled area (i.e., number of subsamples by frame area), yielding standardised densities for each habitat and timepoint. All samples were preserved in 70% ethanol in the field for later identification in the laboratory. Identification of the macroinvertebrates was carried out using a stereo microscope (Nikon SMZ‐2T), following identification keys and literature for macroinvertebrates (Tachet et al. 2010) and for dragonflies (Askew 2004; Bellmann and Helb 2022; Kohl 1998). Taxonomic cross‐validation was conducted by a second taxonomist for a subset of specimens, particularly in cases of uncertainty or ambiguity. Taxa were identified to the highest reliable level. Some were kept at genus or family levels when species identification was impossible, due to immature stages or lack of diagnostic features.

### 2.3 Environmental Sampling

To describe water quality and environmental conditions of rice paddies and wetlands, conductivity (Hach, CDC40105), dissolved oxygen (Hach, LDO10105), pH (Hach, PHC10105), and temperature were measured with a multiparameter device (HACH HQ4300). Water depth was measured in the field with a folding ruler, and the water surface areas of the paddies and ponds were measured using the polygon tool in Google Maps.

Each environmental variable (but surface area) was measured at three points per site, and the means were used for later analysis. If a ditch was present, measurements were also taken at three points along it. The mean of the three measurements was used for analysis. We measured these environmental variables twice a year, in July and August of 2022 and 2023.

### 2.4 Statistical Analyses

Analyses were carried out using R (version 4.4.3, R Core Team 2023) and visualisations created using the *ggplot2* package (Wickham 2016). The general analysis strategy was to first compare environmental variables and macroinvertebrate community data between rice paddies and wetlands, followed by additional comparisons among and within rice paddies to assess the influence of ditches. Physicochemical environmental parameters were compared using univariate and multivariate linear models. Estimates of macroinvertebrate diversity were calculated at the local (alpha) and regional (gamma) scale and compared using generalised linear models. Community composition was compared and linked to environmental variables using constrained ordination (partial distance‐based redundancy analysis—dbRDA). Finally, we used indicator species analyses to identify taxa showing strong associations with a particular habitat. All comparisons are listed in Table S2 and described in more detail below.

#### 2.4.1 Environmental Variables

##### 2.4.1.1 Rice Paddies Versus Wetlands

We compared the environmental parameters conductivity (μS/cm), dissolved oxygen (mg/L), water depth (cm), pH, water temperature (°C), and water surface area (ha) between rice paddies and wetlands using MANOVA. For sites with multiple paddies and for paddies with measurements from centre and ditch we used mean values. All environmental variables were log‐transformed, except for pH (which is already on a logarithmic scale). We conducted a MANOVA to assess whether the six environmental variables collectively differ in multivariate space between the two habitats. Because MANOVA assumptions were not fully met (Tables S3–S6, Figure S1), we relied on Pillai's trace statistic to assess significance, as it is considered most robust to deviations from multivariate normality and homogeneity of covariances (Olson 1976). Multivariate effect size was quantified as eta‐squared (*η* ^2^). To assess which variables contributed to the overall difference, MANOVA was followed by univariate linear mixed‐effects models (LMMs; *lmer* function in the *lme4* package, Bates et al. 2015) for each environmental variable. For all models, we addressed the non‐independence of repeated measurements by adding random effects for sampling sites and sampling periods (year × sampling round). We validated the LMM models using a Satterthwaite Type III test, with *p*‐values adjusted for multiple testing using the false discovery rate (FDR/Benjamini–Hochberg method; Benjamini and Hochberg 1995).

##### 2.4.1.2 Rice Paddies and Ditches

To examine the environmental differences between rice paddies with ditches and those without, we organised the data into three complementary subsets: (i) a centres‐only dataset, which compares the centres of paddies with ditches to those without; (ii) a between‐paddy dataset, where ditch and centre samples from paddies with ditches are combined and compared against centres of paddies without ditches; (iii) a within‐paddy dataset, which directly compares ditch and paddy centre samples within the same paddy. We will refer to these as the ‘centre‐only’, ‘between‐paddy’, and ‘within‐paddy’ datasets.

We used LMMs to test for differences in the environmental variables conductivity, water depth, dissolved oxygen, water temperature, and pH. For the between‐paddy and centre‐only comparison, we considered ditch (present or absent) as a fixed effect, while for comparisons within‐paddies, the position of sampling (paddy centre or ditch) was included as a fixed effect. For all models, we included random effects for the sampling sites and for the sampling periods (year × sampling round). We validated the LMM models using a Satterthwaite Type III test with FDR‐adjusted *p*‐values.

#### 2.4.2 Macroinvertebrate Diversity

##### 2.4.2.1 Rice Paddies Versus Wetlands

To estimate and compare gamma diversity of macroinvertebrate communities in rice paddies and wetlands, we used abundance‐based and sample‐size‐based rarefaction and extrapolation curves, visualised using the *iNEXT* package (Chao et al. 2014; Hsieh et al. 2016). We combined data collected over 2 years and all sampling rounds to evaluate overall diversity throughout the study period. Data from sites with multiple paddies were aggregated at the site level.

We calculated macroinvertebrate densities and taxa richness (alpha diversity) for each sample. Additionally, we computed the Shannon diversity index using the *diversity* function in the *vegan* package (Oksanen et al. 2025).

Species richness was analysed using a Generalised Linear Mixed Model (GLMM) with a Poisson distribution and a log link function (using the *glmer* function in the *lme4* package) after checking for overdispersion via the *DHARMa* package and meeting diagnostic criteria. Log‐transformed densities and Shannon diversity were analysed with LMMs. All models included habitat type as a fixed factor, while sampling site and sampling period (year × sampling round) were treated as random effects. We assessed the significance of fixed effects using Type III ANOVA, with Wald *χ* ^2^ tests for GLMMs (from the *car* package, Fox and Weisberg 2019) and *F*‐tests with Satterthwaite approximation for LMMs.

##### 2.4.2.2 Rice Paddies and Ditches

To evaluate the effect of ditches on macroinvertebrate density, taxon richness (alpha and gamma), and Shannon diversity in rice paddies, we arranged the count data again into three datasets: between‐paddy, centre‐only, and within‐paddy. We fitted models with ‘ditch presence’ as a fixed effect for the between‐paddy and centre‐only comparison, while the ‘position of sampling’ (paddy centre or ditch) was used for the within‐paddy dataset. All models controlled for the random effects of sites and sampling period (year × sampling round).

#### 2.4.3 Community Composition

##### 2.4.3.1 Rice Paddies Versus Wetlands

To compare the taxonomic composition of macroinvertebrate communities in rice paddies and wetland habitats, we used the *vegan* package in R. To address unequal sampling effort, macroinvertebrate counts were standardised to densities (individuals per m^2^) by dividing total counts per sample by the total sampled area. At sites with multiple paddies or sampling positions (e.g., paddy centre and ditch), densities were averaged to obtain a single site‐level estimate. We assessed the association strength of macroinvertebrate taxa with rice paddies and wetlands using the *strassoc* function from the *indicspecies* package (De Cáceres and Legendre 2009) with 1000 bootstrap replicates to determine confidence intervals. The analysis utilised generalised point‐biserial correlation coefficients (*r*_pb_; Borcard et al. 2011; De Cáceres and Legendre 2009; Chytrý et al. 2002). Additionally, we tested the statistical significance of the associations through a permutation (9999 permutations) approach with the *signassoc* function (*indicspecies* package) and *p*‐values adjusted using the Sidak correction for the group‐wise comparison per taxon. We corrected the results against the total tested taxa set using false discovery rate adjustment. Habitat type was used as the grouping factor, utilising a limited permutation scheme that permitted permutations over time (year × sampling round) within each site.

We subsequently conducted a partial dbRDA (Legendre and Andersson 1999; Borcard et al. 2011; Legendre and Marti 2011) using the *dbrda* function from the *vegan* package. This constrained ordination method enabled us to link dissimilarities in macroinvertebrate community composition to environmental variables, including water surface area, conductivity, pH, dissolved oxygen, water temperature, and depth. We also included habitat type as a proxy for structural heterogeneity because quantitative measurements of habitat structure (e.g., vegetation complexity, substrate heterogeneity etc.) were not available, therefore following the example of Borcard et al. (1992) for insufficient quantitative data. We calculated the square root of the absolute densities to minimise the impact of dominant species. Then, a Bray–Curtis dissimilarity matrix was generated using the *vegdist* function, with a correction for negative eigenvalues applied by taking the square root of the Bray–Curtis matrix as described in Legendre and Andersson (1999).

Before performing dbRDA, we tested whether multivariate dispersion was homogeneous across environments, using the *betadisper* function with square‐rooted Bray–Curtis distances, followed by a permutation test (Table S13).

To reduce the chance of overestimating community variation caused by environmental variables, we used a forward selection process for the model. This process employed a double stopping criterion based on adjusted *R* ^2^ and *p*‐values (Blanchet et al. 2008) of the environmental variables. We first performed the dbRDA using a full model that included all explanatory variables: habitat type, water surface area, conductivity, dissolved oxygen, water depth, pH, and temperature. To account for the non‐independence of samples, we controlled for variation from site, year, and sampling round by partialling out the effects with the *Condition* term within *dbrda* before testing the constrained explanatory variables. Following a significant global permutation test of the full model with *anova.cca*, the forward selection of environmental variables was conducted using the *ordiR2step* function, with *p*‐values adjusted using the FDR method. The parameters pH and temperature were excluded, and the final model included the remaining variables: habitat type, water surface area, conductivity, dissolved oxygen and water depth (Table S14). The significance of individual explanatory variables and constrained ordination axes was subsequently assessed using permutation tests with *anova.cca*. For multiple comparisons, *p*‐values were adjusted using the FDR method, while the global model was not corrected, as it represents a single hypothesis test.

All permutation schemes within this analysis were restricted within sites, thereby permuting only across sampling periods (year × sampling round) and set to *n* = 999 unless otherwise mentioned.

##### 2.4.3.2 Rice Paddies and Ditches

To assess the effect of ditches in rice paddies, we compared community composition on three datasets: between‐paddy, centre‐only, and within‐paddy. To identify taxa associated with different structures within the rice paddies, we performed indicator species analyses using the *indicspecies* package. We calculated generalised point‐biserial correlation coefficients (*r*_pb_) and confidence intervals with 1000 bootstrap replicates to evaluate the strength of the associations. Because of the small sample size, the confidence intervals are mainly for guidance (De Cáceres and Legendre 2009). Statistical significance was determined through permutation procedures, with *p*‐values adjusted using the Sidak correction for the group‐wise comparison per taxon. Additionally, we corrected the results against the total tested taxa set by adjusting *p*‐values for the FDR. For the comparisons between paddy and centre‐only samples, we included ‘ditch presence’ as a grouping factor. In the within‐paddy comparisons, we looked at the associations with ‘position in paddy’, specifically the connection between the ditch macroinvertebrate community and the paddy centre community from the same paddies.

## 3 Results

### 3.1 Environmental Variables

#### 3.1.1 Rice Paddies Versus Wetlands

**FIGURE 2:**
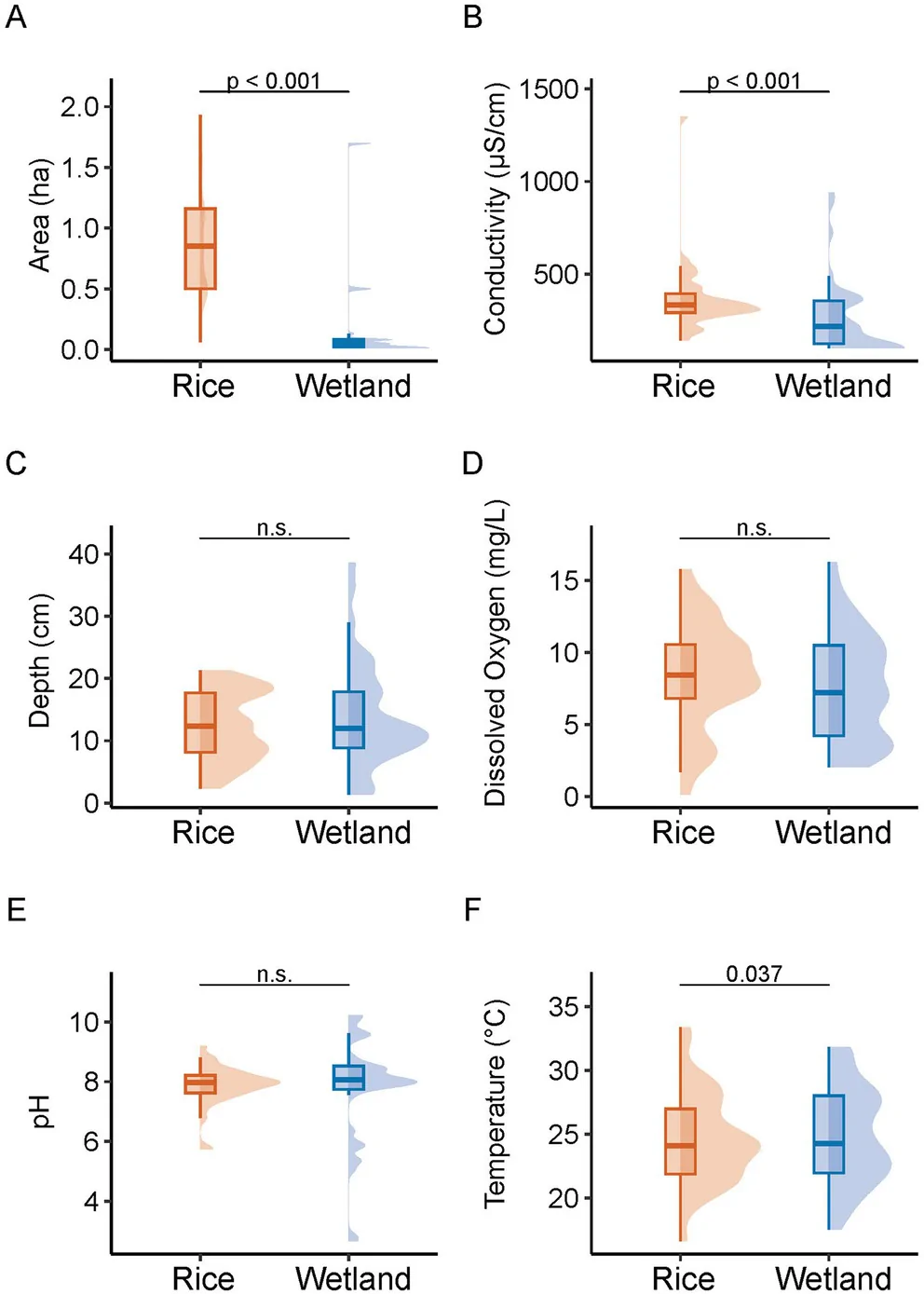
Environmental measurements in rice (red) and wetlands (blue): (A) Water surface area, (B) Conductivity, (C) Water depth, (D) Dissolved oxygen concentration, (E) pH, and (F) Water temperature. Boxplots show the interquartile range with the median line, and whiskers indicate the highest and lowest values, excluding outliers. Projected density plots show the distribution of the data. Significance levels are based on LMM results (Table S8).

The measured environmental variables from rice paddies and wetlands are shown in Figure 2 (detailed measurements for each sampling occasion in Figure S2). MANOVA indicated a significant multivariate effect of habitat type on environmental variables (Table S7; Pillai's Trace = 0.507, *F*(6, 72) = 12.33, *p* < 0.001; effect size *η* ^2^ = 0.51).

The univariate analyses of the environmental variables with LMMs (Tables S8 and S9) showed that rice paddies had higher water surface areas (mean and 95% CI: 0.87 ha [0.68–1.09] vs. 0.19 ha [0.07–0.33]; *F*(1,70.76) = 63.82, *p* < 0.001), higher conductivity (332.91 μS/cm [251.90–439.87] vs. 219.85 μS/cm [166.34–290.48]; *F*(1, 65.68) = 22.02, *p* < 0.001) and slightly lower water temperature (23.78°C [21.19–26.67] vs. 24.98°C [22.27–28.02]; *F*(1, 64.02) = 4.52, *p* = 0.037) compared to wetlands. No significant differences were found in water depth, dissolved oxygen concentration or pH between rice paddies and wetlands.

#### 3.1.2 Rice Paddies and Ditches

**FIGURE 3:**
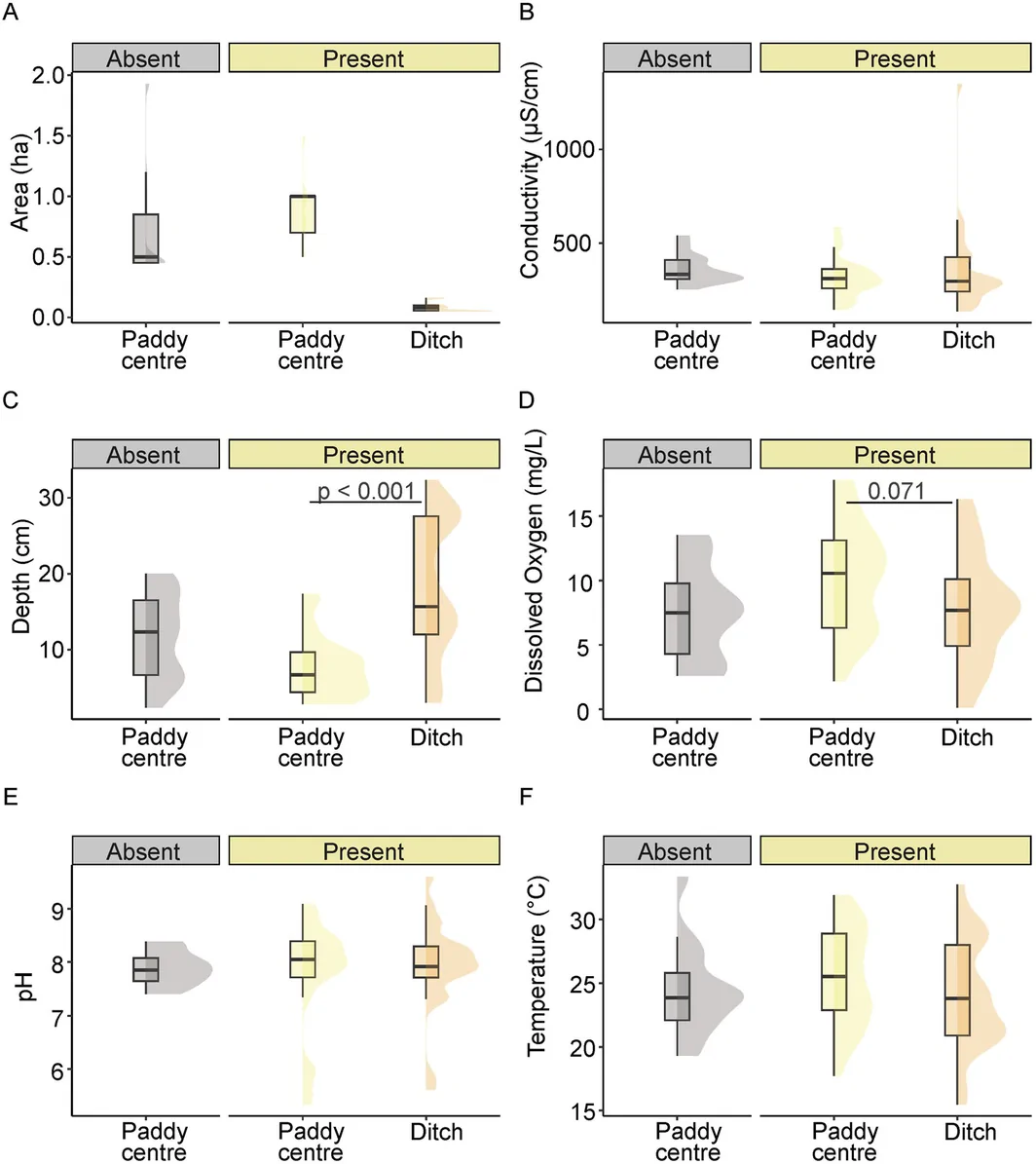
Environmental measurements in paddy centres and ditches of paddies without and with ditches: (A) Water surface area, (B) Conductivity, (C) Water depth, (D) Dissolved oxygen concentration, (E) pH, and (F) Water temperature. Boxplots show the interquartile range with the median line, and whiskers indicate the highest and lowest values, excluding outliers. Projected density plots show the distribution of the data. Significance levels are based on LMM results (Table S9).

The comparison of environmental variables in rice paddies with and without ditches (between‐paddy dataset; Figure 3, Figure S3, Table S10) indicates that, overall, the environmental variables conductivity, water depth, dissolved oxygen, pH, or water temperature were not significantly affected by the presence of a ditch in the merged dataset. Likewise, in the paddy centres (centre‐only dataset), no significant differences were observed between paddies with and without ditches. In paddies with ditches, water depth was significantly higher in the ditch (mean and 95% CI: 15.22 cm [10.51–21.85]) than in paddy centres (6.93 cm [4.63–10.18]; F(1, 39.53) = 19.71, *p* < 0.001). Dissolved oxygen was lower in the ditch (6.64 mg/L [3.77–11.24]) compared to the paddy centres (9.03 mg/L [5.26–15.08]; *F*(1, 38.99) = 5.19, *p* = 0.071), albeit not significantly so.

### 3.2 Macroinvertebrate Diversity

#### 3.2.1 Rice Paddies Versus Wetlands

**FIGURE 4:**
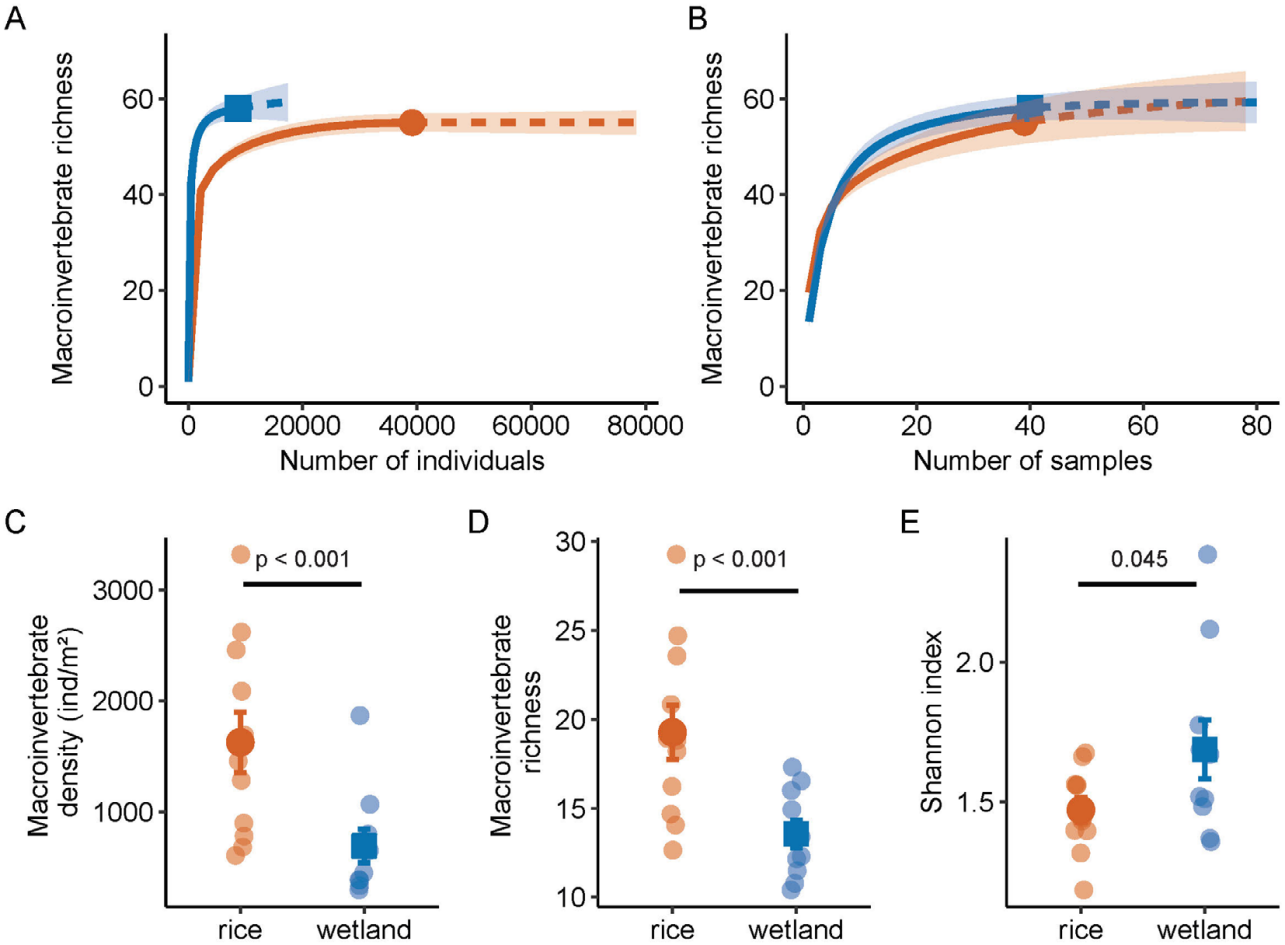
Macroinvertebrate diversity rarefaction and extrapolation curves based on abundance (A) and sample size (B) in rice paddies and wetlands, representing gamma diversity (total macroinvertebrate taxa richness). (C) Mean macroinvertebrate densities (number of individuals/m^2^) per site, calculated from the average across multiple sampling rounds (year × sampling round). Alpha diversity metrics are shown for (D) macroinvertebrate richness and (E) the Shannon index, with coloured dots indicating average values per site derived from mean richness of each sampling event (sampling round × year). All plots with SE.

Rarefaction curves showed that regional (gamma) taxon richness was higher in wetlands with 58 observed taxa and an estimated richness of 60.0 (95% CI: 58.0–68.15) compared to rice paddies, which had 55 observed taxa and an estimated richness of 55.0 (95%: 55.0–59.32; Figure 4A,B), consistent with greater habitat heterogeneity across wetlands. Abundance‐based rarefaction (Figure 4A) showed a clear separation in estimated gamma diversity between wetlands and rice paddies, with non‐overlapping confidence intervals. In contrast, sample‐based rarefaction (Figure 4B) showed broader confidence intervals and partial overlap, indicating that inference depends on the standardisation method. Sample‐based standardisation can introduce greater uncertainty because the sample sizes required to reach equivalent coverage vary across bootstrap replicates, resulting in wider confidence intervals (Chao et al. 2014; Hsieh et al. 2016).

At the local level (alpha diversity), however, taxon richness was significantly higher in rice paddies than in wetlands (*χ* ^2^(1) = 41.38, *p* < 0.001, Figure 4D). In contrast, the Shannon index was higher in wetlands (*F*(1,77) = 4.17, *p* = 0.045, Figure 4E), indicating that wetland samples contained a more even taxon distribution. Densities were substantially higher in rice paddies (1212.2 ind/m^2^ [713.7–2058.5] vs. 447.9 ind/m^2^ [263.3–761.2]; *F*(1,68.15) = 32.28, *p* < 0.001; Figure 4C). These results indicate that wetlands are richer at the regional level (gamma diversity) because sites differ more from one another, while rice paddies have a higher local richness and contain higher densities of macroinvertebrates, but lower evenness—i.e. paddies contain many individuals dominated by a few taxa.

#### 3.2.2 Rice Paddies and Ditches

**FIGURE 5:**
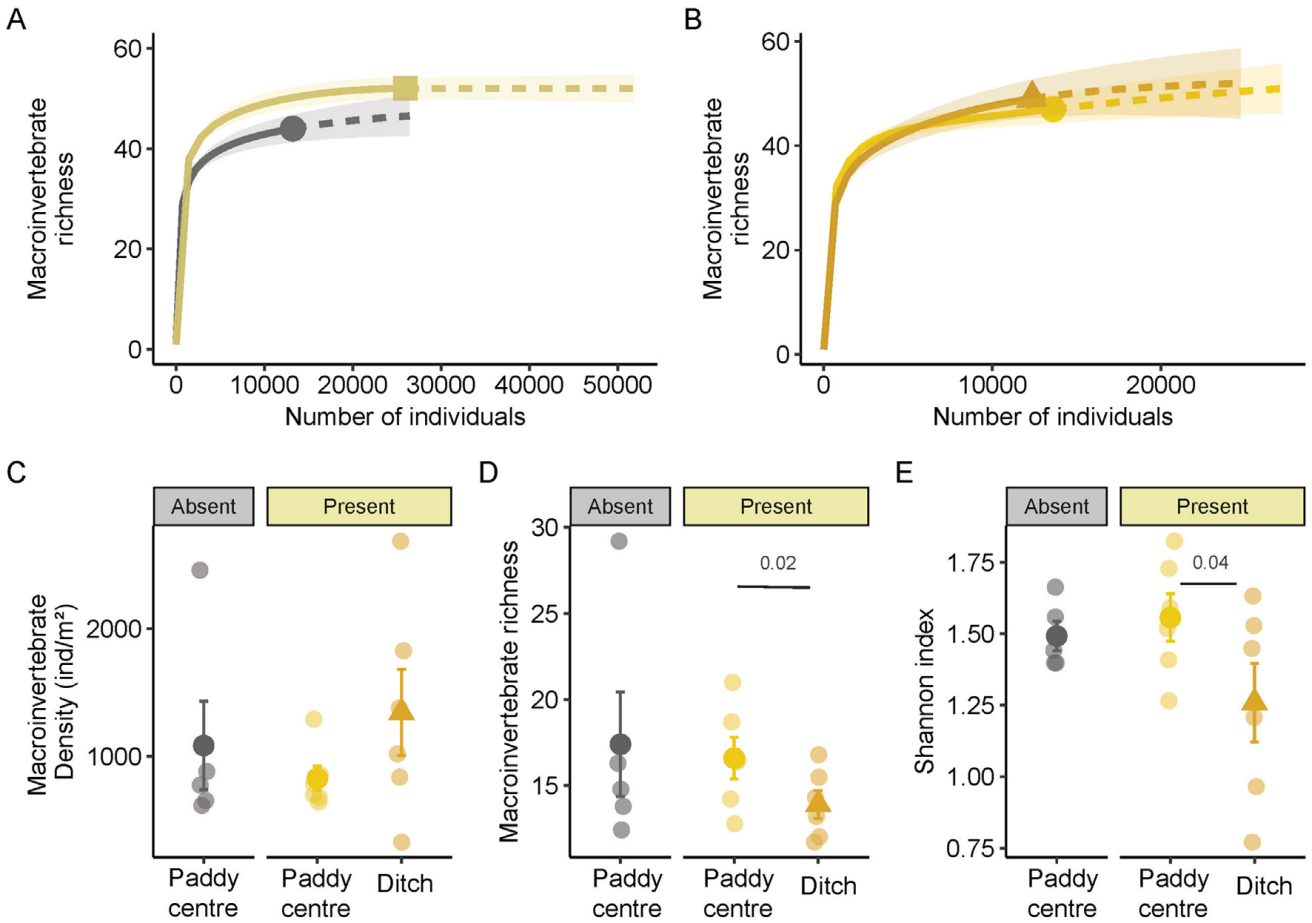
Macroinvertebrate diversity comparison between rice paddies with a ditch present and absent. (A) Abundance‐based rarefaction and extrapolation curve for merged dataset, (B) Abundance‐based rarefaction and extrapolation ditch paddies only dataset. (C) Macroinvertebrate density, (D) Richness and (E) Shannon index. All values with SE.

Rarefaction curves indicated that the sampling captured most of the taxon richness present in rice paddies (Figure 5A,B, Figure S4). Regionally, rice paddies with ditches supported a slightly higher macroinvertebrate richness, with 52 observed taxa (estimate and 95% CI: 52 [52.00–56.65]), compared to rice paddies without ditches, for which only 44 taxa were observed (estimate and 95% CI: 48 [44.00–57.78]) (Figure 5A). This likely results from some turnover of taxa among different ditch‐equipped paddies, since there were no significant differences in macroinvertebrate richness, Shannon diversity, or density between paddies with and without ditches (Figure 5C–E). Abundance‐based rarefaction for the presence of ditches in rice paddies (Figure 5A) shows confidence intervals largely non‐overlapping across the observed range and only overlapping in parts of the extrapolated section. This was consistent whether we compared only macroinvertebrates from the paddy centres or combined the centre and ditch samples for the paddies with ditches (Figure 5C–E, Table 1). Within‐paddy comparisons (Figure 5B) between ditch and centre habitats revealed considerable overlap of confidence intervals across the entire rarefaction curve, with broader uncertainty. Hence, there is no evidence for differences in richness between the paddy centres (47 taxa, estimate: 57.00 [47.00–68.92]) and the ditches (observed: 49 taxa, estimate: 52.60 [49.00–63.36]) on a regional scale. However, within the paddies that contained ditches, the sample position had a significant influence on macroinvertebrate alpha‐diversity (Figure 5C–E). Ditch samples exhibited significantly lower taxon richness and Shannon diversity compared to paddy centres (richness: 13.9 [12.00–16.10] vs. 16.6 [14.40–19.10]; Shannon: 1.19 [0.90–1.49] vs. 1.49 [1.19–1.78]). Densities of macroinvertebrates were higher in ditches (765.00 ind/m^2^ [385.00–1518.00] vs. 575.00 ind/m^2^ [290.00–1142.00]), though this difference was not statistically significant (Table 1).

**TABLE 1:** Macroinvertebrate diversity models (Type III ANOVA) for richness, Shannon diversity, and density in the three rice paddy comparison datasets; included site ID and sampling period as random effects.

Response	Dataset	Predictor	NumDf	DenDf	MS	*F*	*p*
Density	Centre‐only	Ditch presence	1	33.24	0.10	0.27	0.61
	Between‐paddy	Ditch presence	1	9.42	0.12	0.36	0.56
	Within paddy	Position in paddy	1	36.63	0.93	1.64	0.21
Shannon	Centrr‐only	Ditch presence	1	36.00	0.12	0.68	0.42
	Between‐paddy	Ditch presence	1	37.00	< 0.01	< 0.01	1.00
	Within paddy	Position in paddy	1	41.16	0.99	4.57	**0.04**

Response	Dataset	Predictor	Df	*χ* ^2^	*p*
Richness	Center‐only	Ditch presence	1	0.07	0.79
	Between‐paddy	Ditch presence	1	1.86	0.17
	Within paddy	Position in paddy	1	5.37	**0.02**

### 3.3 Community Composition

#### 3.3.1 Rice Paddies Versus Wetlands

**FIGURE 6:**
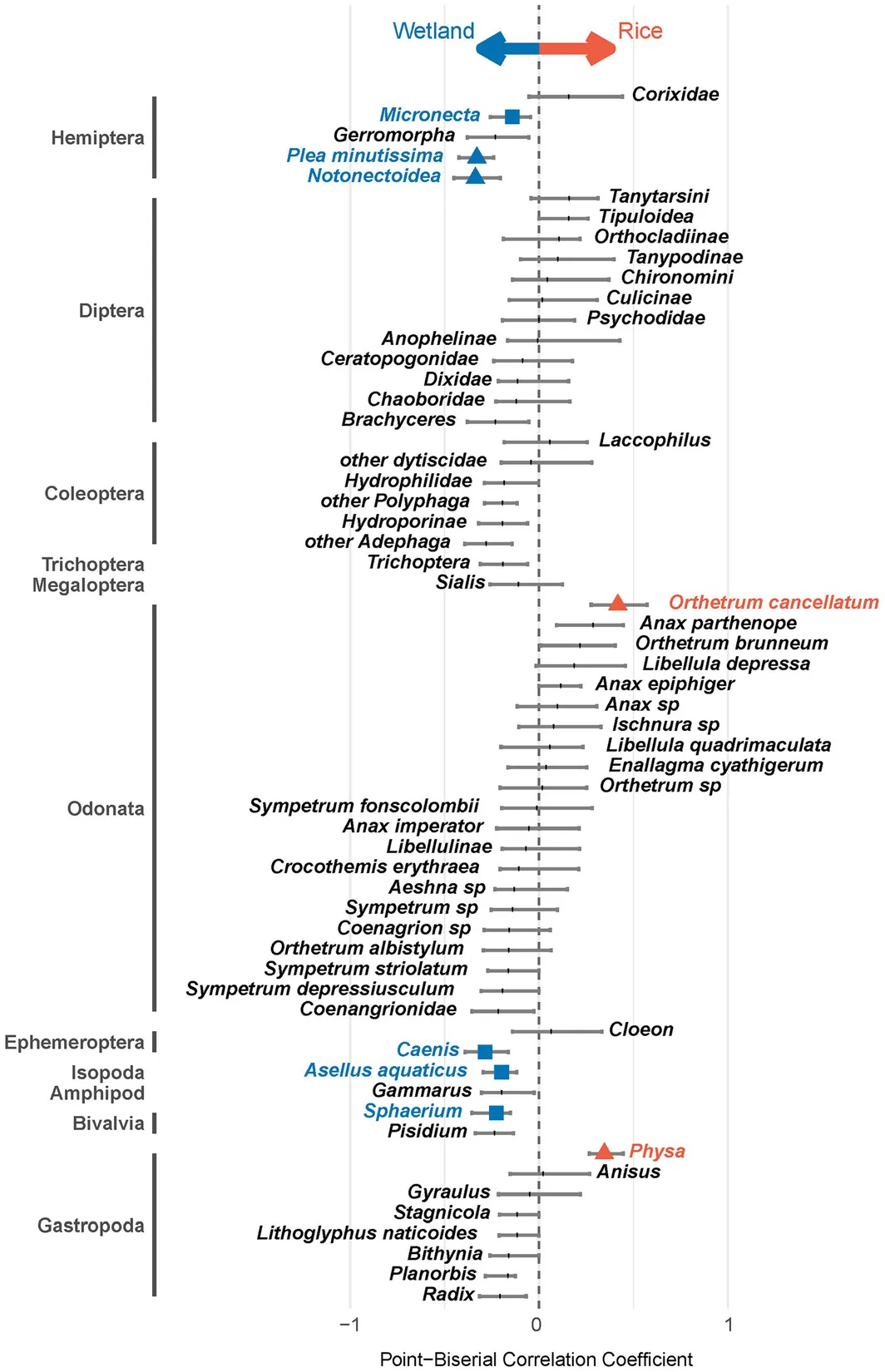
Macroinvertebrate association strength (*r*_pb_) with wetland (blue) and rice paddies (red) habitats, expressed as the point‐biserial correlation coefficient. *p*‐values adjusted with FDR: Square *p* > 0.05, triangles *p* > 0.005. Taxa are displayed at the highest reliable taxonomic resolution achieved during identification. Full results are provided in Table S12.

The associations of macroinvertebrates to rice and wetland habitats varied moderately among taxa (Figure 6, Tables S11 and S12). Since we had a large number of taxa relative to the limited number of sites, only a few taxa remain associated significantly with one habitat after correction for multiple testing. The gastropod genus *Physa* (*r*_pb_ = 0.35, *P*_FDR_ = 0.004) and the dragonfly *Orthetrum cancellatum* (*r*_pb_ = 0.42, *P*_FDR_ = 0.004) were significantly associated with rice paddies. Notonectoidea (*r*_pb_ = 0.34, *P*_FDR_ = 0.006), *Plea minutissima* (*r*_pb_ = 0.33, *P*_FDR_ = 0.004), *Caenis* (*r*_pb_ = 0.29, *P*_FDR_ = 0.045), *Sphaerium* (*r*_pb_ = 0.23, *P*_FDR_ = 0.042), *Asellus aquaticus* (*r*_pb_ = 0.20, *P*_FDR_ = 0.048), and *Micronecta* (*r*_pb_ = 0.14, *P*_FDR_ = 0.036) were significantly associated with wetlands.

**FIGURE 7:**
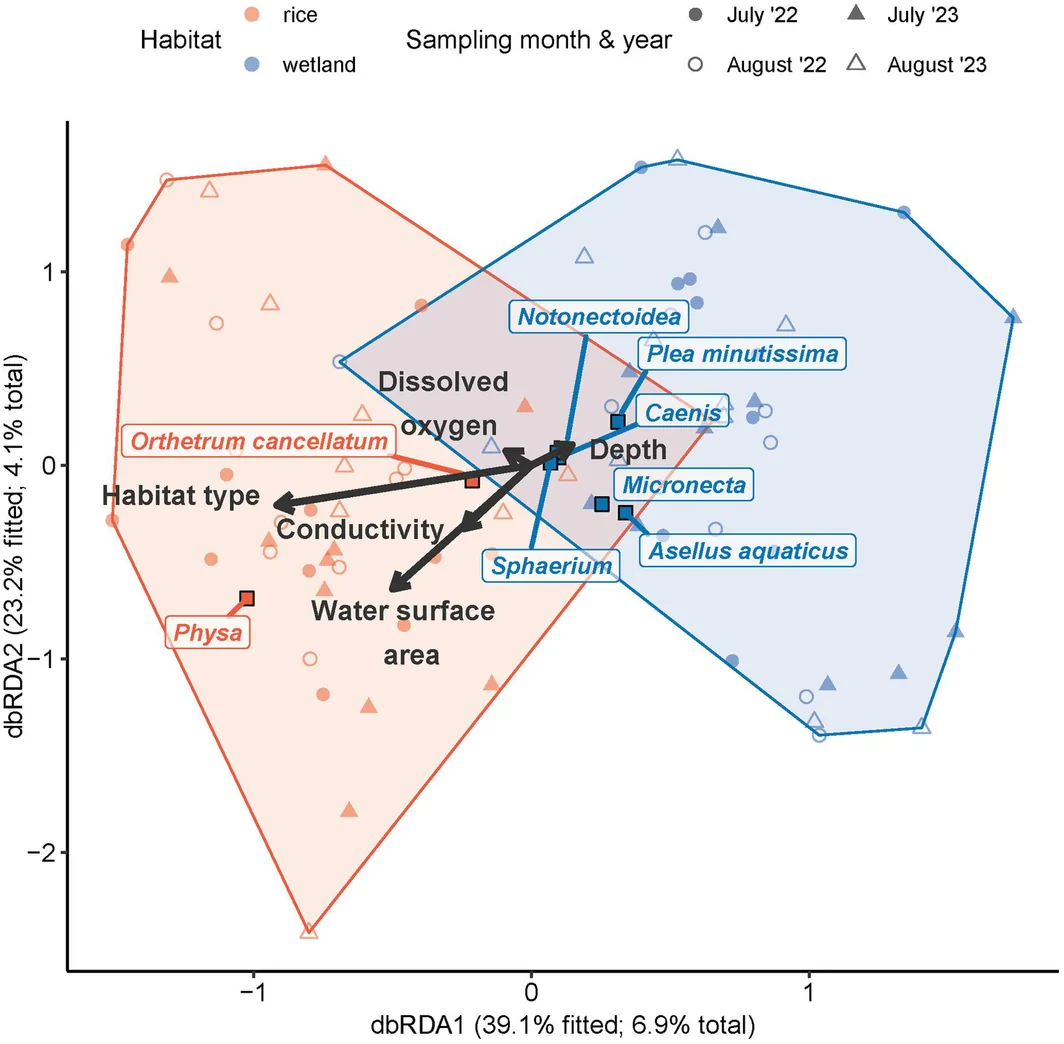
Macroinvertebrate communities in wetlands and rice paddies, based on dbRDA analysis. The shaded convex hulls and colours represent the environments: Rice paddies (red) and wetlands (blue). Symbols indicate samples from different sampling campaigns: Circles for 2022 and triangles for 2023; shaded shapes denote July samples, while unshaded shapes represent August samples. Black arrows display the loadings of environmental variables. Only scores for significant macroinvertebrate taxa associations (FDR *p* < 0.05) are included and marked with coloured squares and labels in red for rice paddies and blue for wetlands. Axis labels show the percentage of variation explained by the constrained model (fitted) and relative to the overall community variation (total).

The analysis of macroinvertebrate composition using dbRDA shows partial separation of rice and wetland communities and indicates that environmental variables significantly influenced their composition (Figure 7, Table 2). Multivariate dispersion differed between habitats, with wetlands (mean = 0.61, SD = 0.046) showing slightly higher within‐group dissimilarity than rice paddies (mean = 0.51, SD = 0.050), which indicates that some of the observed differences in community composition may be influenced by greater heterogeneity among wetland sites rather than solely by differences in average community structure. The environmental variables explained 14% of the total variation, while an additional 21% was accounted for by the conditioned effects of site and sampling period (Table 3). Among the constrained factors, the first three axes of the redundancy analysis (dbRDA1–3, Table S15) were significant and accounted for 82.1% of the constrained variation. Notably, habitat type was identified as the strongest predictor in the dbRDA, followed by water surface area and conductivity, all of which significantly explained variation in macroinvertebrate community composition (Table 2). Dissolved oxygen and water depth were also significantly associated with community composition (Table 2).

**TABLE 2:** Results of dbRDA comparing macroinvertebrate community composition between rice paddies and wetlands. Shown are the results of the overall model, individual environmental variables (predictors), and constrained axes. *p*‐values for predictors and axes were adjusted using FDR correction; the global model test was not corrected.

	df	Sum of squares	*F*	*p*
Model	5	3.69	2.70	**0.001**
***Pedictor***				
Habitat	1	1.37	5.01	**0.002**
Water surface area	1	0.79	2.89	**0.002**
Conductivity	1	0.71	2.60	**0.002**
Dissolved oxygen	1	0.44	1.59	**0.013**
Water depth	1	0.38	1.40	**0.039**
*Axes*				
*dbRDA1*	1	1.44	5.27	**0.002**
*dbRDA2*	1	0.86	3.13	**0.002**
*dbRDA3*	1	0.73	2.66	**0.002**
*dbRDA4*	1	0.35	1.29	0.237
*dbRDA5*	1	0.31	1.13	0.237
Residual	63	17.23		R2: 0.10

*Note:* Bold values indicate statistically significant results (*p* < 0.05).

**TABLE 3:** Partitioning of variation (inertia) in the dbRDA model comparing rice paddy and wetland macroinvertebrate communities. Total inertia represents overall variation in community composition. Conditioned inertia corresponds to variation attributed to site, year, and sampling round (partialled out in the model). Constrained inertia represents variation explained by habitat type and selected environmental variables. Unconstrained inertia represents residual variation not explained by the model.

	Inertia	Proportion
Total	26.57	1.00
Conditioned	5.65	0.21
Constrained	3.69	0.14
Unconstrained	17.23	0.65

#### 3.3.2 Rice Paddies and Ditches

**FIGURE 8:**
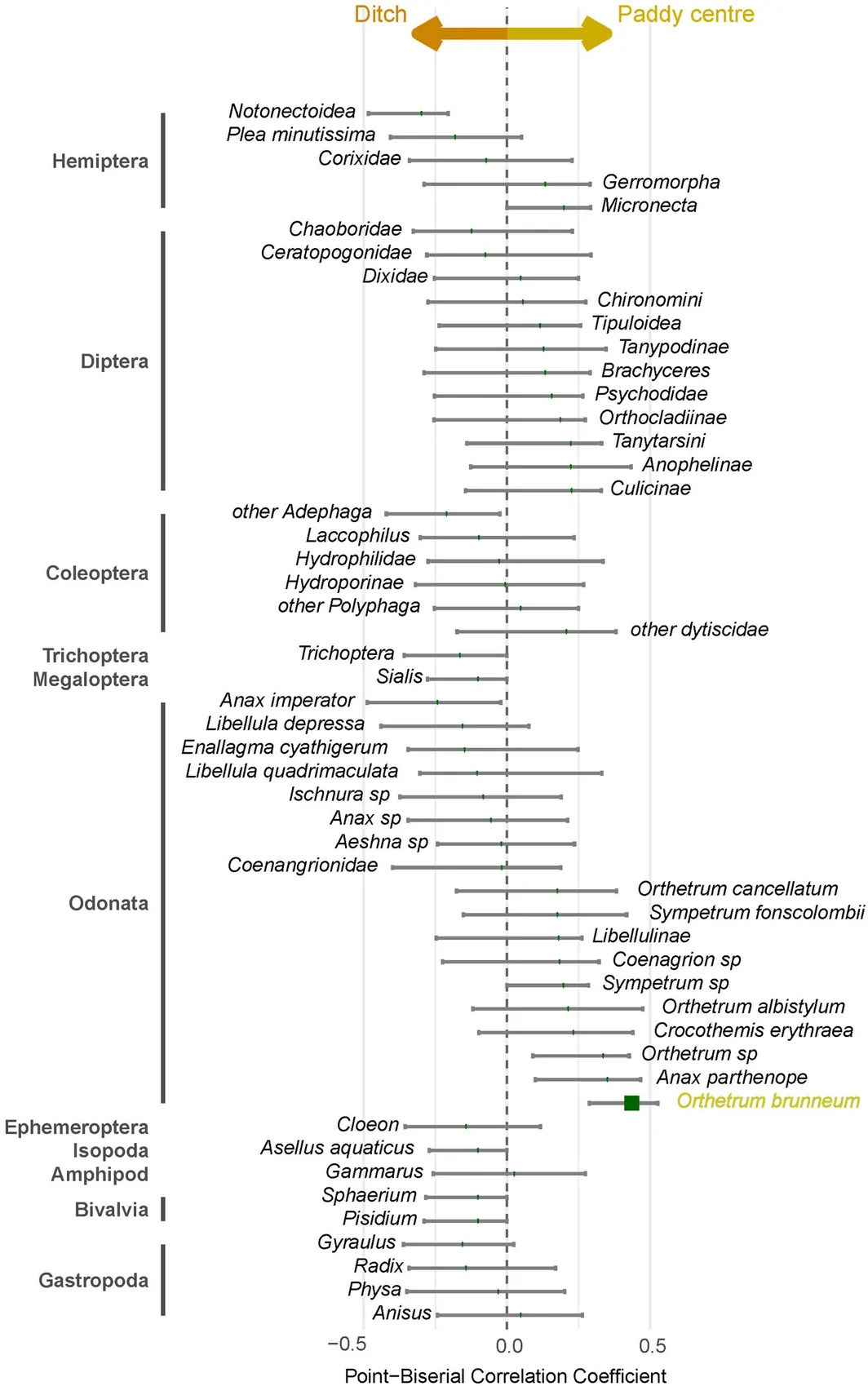
Macroinvertebrate association strength (*r*_pb_) within paddies in the ditches (yellow, negative *r*_pb_) and paddy centres (orange, positive *r*_pb_). *p*‐values adjusted with FDR: Triangles *p* = 0.01. Taxa are displayed at the highest reliable taxonomic resolution achieved during identification. Full results are provided in Table S16.

The impact of ditches on macroinvertebrate associations in rice paddies was generally weak (Figure 8, Figures S5 and S6, Tables S16–S18). Point‐biserial correlation coefficients (*r*_pb_) ranged from −0.29 to 0.27 between paddies with and without ditches, and from −0.35 to 0.39 between ditches and centres within paddies possessing a ditch, indicating that strong habitat specialisation was rare in our dataset. After correction for multiple testing, only the dragonfly species *Orthetrum brunneum* was significantly associated with paddy centres (*r*_pb_ = 0.39, *P*_FDR_ = 0.01). Interestingly, the association strength of macroinvertebrate taxa was positively correlated between the wetland‐rice paddy comparison and the within‐paddy comparison between ditch and paddy centre (Pearson's *r* = 0.35, 95% CI = 0.08–0.57, *p* = 0.011; Figure S7), indicating that several taxa associated with wetlands also tended to be associated with ditches, including freshwater bivalves (e.g., *Sphaerium*, *Pisidium*), hemipterans ( *Plea minutissima* , Notonectidea) and aquatic Coleoptera (Adephaga). Conversely, taxa associated with rice paddies were generally more strongly linked to paddy centres, including odonate nymphs such as *O. brunneum* , *O. cancellatum* , and *Anax parthenope*, as well as dipteran larvae with weaker association strengths (e.g., Tipuloidea, Tanypodinae, Orthocladiinae, Tanytarsini).

## 4 Discussion

Our study is the first to systematically evaluate the aquatic macroinvertebrate communities of recently established rice paddies in Switzerland, which are characterised by a cool‐temperate climate and pesticide‐free management. We had hypothesised that compared to natural wetlands in their vicinity, rice paddies would contain distinct and spatially more homogeneous macroinvertebrate communities characteristic of the environmental conditions in this special habitat. This prediction was largely supported. There was a partial but marked separation in the taxonomic composition of macroinvertebrates from rice paddies and wetlands, with higher dispersion in wetlands, and at the regional scale, wetlands supported a slightly higher number of taxa. At the local scale, on the other hand, rice paddies supported higher macroinvertebrate densities and taxonomic richness, yet their communities showed a lower Shannon diversity (evenness) than those of wetlands. This pattern indicates that the environmental conditions in rice paddies favour a subset of taxa that are well adapted to temporary, nutrient‐rich habitats, whereas wetlands support taxa with a broader range of ecological strategies.

Similar patterns of high local macroinvertebrate richness and density have been observed in Mediterranean rice fields (Leitão et al. 2007; Lupi et al. 2013, 2014) and in France (Rosecchi et al. 2006; Suhling et al. 2000), where rice paddies support diverse aquatic communities, although community evenness remains low due to dominance by few taxa. In Italian rice fields, Lupi et al. (2013) found that benthic macroinvertebrate communities were shaped by water management and habitat structure, echoing our results on the influence of hydroperiod and ditch presence. Rosecchi et al. (2006) reported that conventional rice fields in the Rhône Delta, France, host distinct macroinvertebrate assemblages, with environmental homogeneity leading to lower regional diversity, consistent with our findings in Swiss paddies.

Studies from outside Europe have similarly demonstrated that rice paddies and adjacent wetlands support distinct aquatic macroinvertebrate communities, with temporary‐water specialists dominating paddies and permanent‐water specialists (e.g., Micronecta spp., Notonectidae and Pleidae) being more abundant in permanent wetlands (Choe et al. 2016; Melo et al. 2015; Tawa et al. 2024; Usio and Miyashita 2014; Watanabe et al. 2025). Comparative studies from South America (Pires et al. 2015) report similar patterns. Together, these studies suggest that despite the atypical setting of Swiss paddy rice cultivation compared to more traditional rice‐growing regions, the community differences observed in Swiss rice paddies reflect broader ecological processes operating across geographic regions and cultivation practices. Overall, our results contribute to the evidence that rice paddies host distinct macroinvertebrate communities compared with natural wetlands and contribute substantially to aquatic biodiversity in agricultural landscapes. The key habitat characteristics separating the rice paddies from the wetlands were their larger surface area and higher water conductivity. This reflects the intentional design of rice paddies as large, open cultivation units, similar to those in more traditional rice cropping regions, where regulated flooding and fertilisation create nutrient‐rich and relatively homogeneous conditions (Bo et al. 2025; Odum 1997; Lawler 2001; Roger and Watanabe 1985). These conditions likely contribute to the differences in macroinvertebrate community structure observed between rice paddies and natural wetlands. However, the chosen environmental variables accounted for only a small part of the overall community variation (14%), indicating that other factors—such as vegetation structure, dispersal mechanisms, biotic interactions, or other unmeasured habitat features—also play a significant role in shaping community assembly. These environmental features also set rice paddies apart from other types of constructed aquatic habitats in Switzerland. Unlike rice paddies, which are large, open, and relatively uniform flooded fields used for agriculture, most new aquatic habitats in Switzerland tend to be small, structurally varied, and often shaded ponds built mainly for amphibian conservation (usually around 100 m^2^; Moor et al. 2022; Fahy et al. 2025).

Habitat type, used here as a proxy for differences in structural heterogeneity between rice paddies and wetlands, was the most important predictor of community dissimilarity, and the indicator species analysis identified taxa showing associations with either rice paddies or wetlands. However, only a limited number of taxa remained significantly associated with one habitat after adjusting for multiple testing, suggesting that habitat associations were generally moderate rather than indicating strong habitat specialisation in most taxa. In part this also reflects limited statistical power, as we compared many taxa collected from only 11 rice paddies and wetlands. This cannot be improved at the moment, as they represent all the currently existing rice paddies in Switzerland. Taxa associated significantly with rice paddies included *O. cancellatum* (Libellulidae; Linnaeus, 1758), a dragonfly, and *Physa* sp. (Physidae), a freshwater snail. Both taxa are tolerant of fluctuating water levels and eutrophic conditions (Leitão et al. 2007; Lupi et al. 2013). *Orthetrum cancellatum* may further benefit from its preference for muddy substrates and its ability to survive short periods of desiccation (Buczyńska and Buczyński 2019; Schnapauff et al. 2000; Wildermuth and Martens 2018). The strong association of *Physa* sp. to rice paddies is likely due to its capacity to exploit several dispersal vectors, including waterbirds and large mammals, which enables rapid colonialisation and high gene flow across habitats (Van Leeuwen et al. 2013). *Physa* sp. can further survive extended periods of desiccation through aestivation (Strachan et al. 2014), making it well suited to the ephemeral conditions of rice paddies.

In contrast, the taxa associated with wetlands are likely requiring stable water conditions and higher structural habitat complexity. They included the superfamily Notonectoidea and *Plea minutissima* (aquatic bugs—Pleidae, Leach, 1817), *Caenis* sp. (a mayfly—Caenidae), *Asellus aquaticus* (an aquatic isopod—Asellidae, Linnaeus, 1758), and two freshwater clams *Pisidium* sp. and *Sphaerium* sp. (Sphaeriidae; Aydin and Camur‐Elipek 2022; Pires et al. 2015; Suhling et al. 2000). The association of the aquatic bugs with natural wetlands was, at first glance, surprising. *Ilyocoris cimicoides* (Naucoridae, Linnaeus, 1758), *Notonecta glauca* (Notonectidae, Linnaeus, 1758) both categorised here within superfamily Notonectoidea, and *P. minutissima* are known to prefer stagnant and slow‐moving waters rich in nutrients and aquatic plants (Aukema and Hermes 2002; Stoianova and Simov 2025), conditions also found in rice paddies. At least for the effectively flightless *P. minutissima* , occurrence might have been limited by colonisation. Adult *I. cimicoides* and *N. glauca* , on the other hand, are good flyers. Hence we can only speculate that other factors than dispersal and unknown habitat requirements may contribute to their lower occurrence in rice paddies.

Overall, these findings imply that rice paddies cannot fully substitute for natural wetlands, but they can provide substantial amounts of aquatic habitat that complement natural wetlands, thereby helping to increase local density and taxonomic richness of aquatic invertebrates in the otherwise depleted Swiss wetland landscape (Gimmi et al. 2011). These insights improve our understanding of rice paddies as a land‐sharing conservation approach in temperate Europe, where wetland loss is drastic, and rice cultivation is becoming more common (Jacot et al. 2018).

Some of the rice paddies we studied had ditches at their sides that were connected to the paddies. The main difference in abiotic conditions provided by these ditches was their greater depth and slightly lower dissolved oxygen concentrations. These ditches can hold water for extended periods after the paddy centres are drained for harvest and—depending on precipitation—may be submerged intermittently. We had hypothesised that ditches could provide suitable habitat for additional taxa, such that paddies containing ditches would support distinct macroinvertebrate communities compared to paddies without ditches and thus contribute to diversity within the rice‐paddy system. This hypothesis did not receive strong support from our data. The influence of ditches on macroinvertebrate communities in rice paddies was, at best, limited. This mirrors the results from a study in Italian rice paddies, where the value of ditches was investigated specifically for dragonflies (Giuliano and Bogliani 2019).

This is not to say that ditches contained the same macroinvertebrate communities as the rest of the rice paddies. In the paddies with ditches, samples from the ditches showed a significantly lower taxon richness and Shannon diversity and somewhat higher macroinvertebrate densities than samples from the centre of the paddies. The ditches are small compared to the adjacent paddies, though, such that their influence did not translate to any significant differences when comparing between paddies with and without ditches.

To identify taxa preferentially associated with either ditches or paddy centres, we also relied on indicator species analysis. Owing to the low number of comparisons possible (six out of 11 paddies had ditches) and the necessity to correct for multiple testing, this analysis found almost no statistically significant associations. Consequently, interpretations regarding the ecological role of ditches should be viewed with caution. The only taxon showing a significant association with paddy centres was the fast‐developing dragonfly *Orthetrum brunneum* (Libellulidae, Fonscolombe, 1837), which has a larval period of only 75 days in Swiss rice paddies (Hoess 2019). The taxa showing relatively strong (but not statistically significant) associations with ditches contained several that were also associated with wetlands, including the freshwater isopod *A. aquaticus* , the freshwater clams *Pisidium* sp. and *Sphaerium* sp., the freshwater snail *Radix* sp. (Lymnaeidae) as well as the predatory water bugs (Notonectoidea and *P. minutissima* ). Indeed, when we compared the taxa's association strengths in the rice paddy versus wetland analysis with those in the paddy centre versus ditch analysis, we observed a significant correlation (Figure S7). The taxonomic composition of ditches showed a greater similarity to wetland‐associated taxa than the paddy centres, suggesting that ditches may provide habitat conditions favourable to some wetland‐associated species. Apart from their functional role in rice cultivation (warming irrigation water, nutrient buffering, pollution control; Liu et al. 2025), ditches can therefore also play an ecological role in providing habitat for species associated with less ephemeral habitats, but at the scale of entire fields, their contribution to the aquatic biodiversity supported by rice paddies remains limited.

Several limitations should be considered when interpreting our results. First, sampling was restricted to July and August and therefore does not capture the complete seasonal community dynamic outside the late flooded cultivation period. Secondly, while this study included all currently existing rice paddies in Switzerland, the number of independent sites remained limited, reducing the power to detect weak ecological effects. Finally, several potentially important drivers of community composition, such as detailed data on fertiliser type and dosage, precise water management schedules, vegetation structure, or landscape connectivity, were not quantified. Future studies incorporating these factors and long‐term monitoring would improve our understanding of the mechanisms underlying community assembly in rice agroecosystem and their contribution to aquatic biodiversity in agricultural landscapes.

## 5 Conclusion

Rice paddies are a new and still rare feature in the Swiss agricultural landscape. Due to their large size, they nevertheless contribute a substantial amount of aquatic habitat to areas where most natural wetlands have been drained. They are suitable primarily for species adapted to waterbodies with short hydroperiods (Pérez‐Méndez et al. 2025; Wellborn et al. 1996; Wiggins et al. 1980), which is the type of wetland that has declined most strongly in Switzerland due to river channelisation and land use change (Gimmi et al. 2011; Schmidt et al. 2015). While they cannot replace natural wetlands (Melo et al. 2015), which maintain higher regional macroinvertebrate diversity and support distinct taxa, we found that under the current climate and pesticide‐free management in Switzerland, rice paddies support a high abundance and a high local diversity of aquatic macroinvertebrates. Biodiversity conservation in rice paddies can therefore be viewed as a successful land‐sharing approach (Augustiny et al. 2025; Echeverría‐Progulakis et al. 2026; Katayama et al. 2019, 2023; Kremen 2015). This perspective is particularly relevant because aquatic habitats are rarely considered in the land sharing–land sparing debate (Hartel et al. 2020). Increasing evidence suggests that aquatic and terrestrial habitats are strongly interconnected (Soininen et al. 2015) and that biodiversity conservation depends on maintaining diverse landscapes that include aquatic ecosystems (Davies et al. 2016; Heim et al. 2018; Tscharntke et al. 2025; Twining et al. 2018). Rice paddies thus represent a valuable component of biodiversity‐friendly agricultural landscapes and may help mitigate the loss of natural temporary wetlands in Switzerland.

## Author Contributions

**Thea Bulas:** conceptualization (lead), data curation (lead), formal analysis (lead), investigation (lead), methodology (lead), project administration (equal), resources (lead), software (lead), validation (lead), visualization (lead), writing – original draft (lead), writing – review and editing (lead). **Benedikt R. Schmidt:** conceptualization (equal), formal analysis (equal), investigation (equal), methodology (equal), supervision (lead), writing – original draft (equal), writing – review and editing (equal). **Christoph Vorburger:** conceptualization (equal), formal analysis (equal), investigation (equal), methodology (equal), supervision (lead), writing – original draft (equal), writing – review and editing (equal). **Roel D'Haese:** investigation (supporting), writing – review and editing (supporting). **Yvonne Fabian:** project administration (lead), resources (equal), supervision (equal), writing – review and editing (equal).

## Funding

This work was supported by Canton of Valais (655024400), Schweizerischer Nationalfonds zur Förderung der Wissenschaftlichen Forschung, Bridge Discovery (grant no. 40B2‐0_203536/1), Canton of Vaud (655024392), Kanton Bern (655034398), Canton of Aargau (4600029524‐10), Ernst Göhner Stiftung (Nassreisanbau_2020‐1960/3), Canton de Fribourg (655024292).

## Conflicts of Interest

The authors declare no conflicts of interest.

## Supporting information


**Table S1:** Characteristics of the sampled rice paddies and wetlands. For hydroperiod of wetlands in the brackets: T—temporary wetland, P—permanent wetland. Lower table are the wetland characteristics of the studied sites.
**Table S2:** Overview of response variables, statistical models, explanatory variables, and random effects used within the study.
**Table S3:** The environmental variables measured in rice paddies and wetlands are presented with sample size (*N*), means, and standard deviation (SD). Results of the univariate normality with Shapiro–Wilk test (*W*‐statistic) are also included. The *p*‐values for each group and dependent variable combination are significant, indicating that the assumption of multivariate normality is not met.
**Table S4:** Multivariate normality assessed with Mardia's test, in addition to the Shapiro–Wilk test. The skewness test showed significance (*p* < 0.05), indicating asymmetrical data distribution. The kurtosis test also indicated significant results, revealing heavy tails or outliers, therefore we tested further for outliers (Mahalanobis distance) and covariance homogeneity (Box's *M* test).
**Table S5:** Box's *M* test.
**Table S6:** Results of Levene's test. Because the results of Box's *M* test were significant, meaning covariance is not homogeneous and the assumption violated, we tested the homogeneity of variance for each dependent variable individually with this test. Conductivity and pH have heterogeneous variance, as indicated by the significant *p*‐values. The non‐significant environmental variables follow the homogeneity of variance.
**Table S7:** MANOVA Univariate ANOVA result table of the environmental parameters, with 1 excluded Outlier, detected with Mahalanobis distances check. Habitat categories were rice paddies and wetlands.
**Table S8:** Comparison of environmental variables between rice paddies and wetlands using linear mixed‐effects models (LMMs). Habitat type (rice paddy vs. wetland) was included as a fixed effect. Random effects accounting for repeated sampling (Site ID and Sampling Campaign) were included for all variables except water surface area, for which only Site ID was used because area did not vary over time. To ensure comparability with the MANOVA results, the same outlier identified by the Mahalanobis distance test was excluded from all LMMs. Marg. *R* ^2^ = marginal *R* ^2^; Cond. *R* ^2^ = conditional *R* ^2^.
**Figure S1:** Correlation of environmental variables in rice paddies (red) and wetlands (blue). Upper panel shows Pearson's correlation with si9gnificance level (asterisks) for overall (grey) and per habitat. Lower plots show pairwise scatterplots of environmental variables per habitat. In the middle are density plots per habitat. All variables but pH were log+1 transformed prior.
**Table S9:** Comparison of environmental variables between rice paddies and wetlands based on linear mixed‐effects models (LMMs). Habitat type was included as a fixed effect, with Site ID and Sampling Campaign as random effects (except for water surface area, which included Site ID only). (F‐) Statistic and *p*‐values refer to the effect of habitat type.
**Figure S2:** Environmental variables for rice paddies and wetlands over time (e.g., year and sampling round, such as 1—July or 2—August).
**Figure S3:** Summary of environmental measurements in paddy centres and ditches (side) in time.
**Table S10:** Differences in environmental variables among rice paddies with and without ditches based on linear mixed‐effects models (LMMs). Ditch presence (present vs. absent) was included as a fixed effect. *p*‐values for the fixed effects were adjusted using the false discovery rate (Benjamini–Hochberg) method.
**Figure S4:** Rarefaction curves for the comparison between rice paddies with and without ditches, and within paddies with a ditch present. Results of the comparison between paddies are shown in A and B; results of the Centre only dataset are shown in C and D; and results of the comparison within paddies with ditches are shown in E and F. Abundance‐based rarefaction is shown in A, C and E, and sample‐size‐based rarefaction in B, D and F.
**Table S11:** Macroinvertebrates counts in 11 rice paddies and 11 wetlands over the years 2022–2023.
**Table S12:** Taxon association strength of macroinvertebrates to the habitat types, rice paddy and wetland. Given are the point biserial correlation coefficient (rpb) and FDR corrected *p*‐values.
**Table S13:** Result of permutation test of dispersion with betadisper() and below the mean & SD per habitat.
**Table S14:** Results of forward selection of explanatory variables with adjusted *R* ^2^ values for multiple testing. The initial full model included macroinvertebrate dissimilarity as a function of habitat, surface area, conductivity, pH, dissolved oxygen, temperature, water depth, and partialled out condition (site ID, year, sampling month). A stopping criterion of *R* ^2^ = 0.101 equal to the full model was applied. The final model retained habitat, surface area, conductivity, dissolved oxygen, water depth, and excluding pH and temperature.
**Table S15:** Eigenvalues and the proportion of constrained and total variation explained by each dbRDA axis for the comparison between rice paddies and wetlands. The last column shows the cumulative percentage of explained variation for the constrained axis.
**Figure S5:** Association strength (rpb) of macroinvertebrates overall in rice paddies (Merged dataset) with and without ditch present Confidence intervals by bootstrap (*n* = 1000). *p*‐values were initially computed using permutation methods and corrected with the default Sidak method, accounting for two group comparisons per species. To control for the total number of taxa tested we adjusted the *p* values with Benjamini–Hochberg method for false discovery rate, but no taxa were significant.
**Table S16:** Association strength for Ditch paddies only dataset.
**Table S17:** Association strength for Centre only dataset.
**Table S18:** Association strength for the between paddies (Centers+ Ditches) dataset.
**Figure S6:** Correlation of habitat association strength (rpb) for macroinvertebrate taxa between wetlands and rice paddies (x‐axis) and within rice paddies between ditch and paddy centre (*y*‐axis). Each point represents one taxon's association strength. Dashed vertical and horizontal lines indicate rpb = 0, separating positive associations with rice paddies or paddy centres from negative associations with wetland and ditch communities. A significant positive Pearson correlation was detected between the two compared datasets (*r* = 0.35, *p* = 0.011, conf *n* = 52 taxa), indicating consistent habitat associations of taxa across rice paddies and wetland habitats. Taxa are labelled using abbreviations derived from the first two letters of each word for multi‐word taxa and the first four letters for single‐word taxa (Taxa list provided in Tables S15–17, and for Hydr1 = Hydrophilidae; Hydr2 = Hydroporinae; Tany1 = Tanypodinae; Tany2 = Tanytarsini). The lower, left blue quadrant displays the taxa associated to Ditch and Wetland areas.

## Data Availability

The data and R scripts supporting the findings of this study are publicly available in Zenodo: Bulas, T. (2026). R scripts and supporting data for ‘Pesticide‐free rice paddies promote diverse and distinct aquatic invertebrate communities in cool‐temperate agroecosystems’ (version v1.0.0). https://doi.org/10.5281/zenodo.21824250.
