# Supplementary material for: Pesticide‐Free Rice Paddies Promote Diverse and Distinct Aquatic Invertebrate Communities in Cool‐Temperate Agroecosystems
Source: Ecol Evol. 2026 Sep 18;16(9):e74287. doi: 10.1002/ece3.74287 (PMC13586779; doi:10.1002/ece3.74287)
Supplement: Supplementary file 1 [file ECE3-16-e74287-s001.docx]

Supplement

***Pesticide-free rice paddies promote diverse and distinct aquatic invertebrate communities in cool-temperate agroecosystems***

**Thea Bulas^,^ Benedikt R. Schmidt, Christoph Vorburger, Roel D'Haese, Yvonne Fabian**

Table S 1 Characteristics of the sampled rice paddies and wetlands. For hydroperiod of wetlands in the brackets: T – temporary wetland, P – permanent wetland. Lower table are the wetland characteristics of the studied sites

| **Site** | **Rice paddy GPS** | **Paddy centre area (ha)** | **Ditch area (ha)** | **Ditch present** | **Paddies sampled** | **Wetland area (ha)** | **Distance to wetland (m)** | **Wetland GPS** | **Hydroperiod** | **River connection** | **Fish present** |
| --- | --- | --- | --- | --- | --- | --- | --- | --- | --- | --- | --- |
| **Brugg** | 47°29'40.4"N 8°14'03.4"E | 1.00 | 0.10 | Yes | 1 of 1 | 0.08 | 595.00 | 47°29'21.7"N 8°13'50.7"E | Temporary | No | No |
| **Detligen** | 46°58'18.6"N 7°15'51.2"E | 0.50 | 0.05 | Yes | 1 of 1 | 0.50 | 350.00 | 46°58'29.7"N 7°15'36.1"E | Permanent | Yes (Aare) | Yes |
| **Jonen** | 47°18'35.9"N 8°23'04.7"E | 0.70 | 0.06 | Yes | 1 of 1 | 0.02 | 246.00 | 47°18'28.7"N 8°22'51.3"E | Temporary | No | No |
| **La Sauge** | 46°58'10.7"N 7°03'43.0"E | 0.40, 0.40, 1.20 | – | No | 3 of 6 | 0.02 | 820.00 | 46°58'30.4"N 7°03'12.5"E | Permanent | No | No |
| **Mühlau** | 47°14'20.3"N 8°23'50.6"E | 0.50, 0.50 | 0.08, 0.08 | Yes | 2 of 2 | 0.05 | 413.00 | 47°14'08.7"N 8°24'07.8"E | Temporary | No | No |
| **Stetten** | 47°23'33.3"N 8°18'30.4"E | 0.45 | – | No | 1 of 1 | 0.03 | 145.00 | 47°23'39.6"N 8°18'33.2"E | Temporary | No | No |
| **Untersiggenthal Ost** | 47°29'40.8"N 8°15'29.6"E | 0.80 | 0.08 | Yes | 1 of 1 | 0.01 | 372.00 | 47°29'46.6"N 8°15'43.2"E | Temporary | No | No |
| **Untersiggenthal West** | 47°29'53.0"N 8°14'39.0"E | 0.50 | – | No | 1 of 1 | 0.01 | 423.00 | 47°30'02.4"N 8°14'21.7"E | Temporary | No | No |
| **Vionnaz** | 46°18'48.3"N 6°55'07.2"E | 1.50 | 0.08 | Yes | 1 of 1 | 0.05 | 1200.00 | 46°18'07.7"N 6°54'43.2"E | Permanent | Yes (Fosse des Talons) | Yes |
| **Witzwil** | 46°58'34.4"N 7°05'02.0"E | 0.45 | – | No | 1 of 1 | 1.70 | 1500.00 | 46°59'27.0"N 7°04'53.2"E | Permanent | No | Yes |
| **Würenlingen** | 47°32'25.9"N 8°14'56.5"E | 1.20 | – | No | 1 of 1 | 0.08 | 461.00 | 47°32'14.0"N 8°15'09.8"E | Permanent | No | Yes |

## Statistical analysis

Table S 2 Overview of response variables, statistical models, explanatory variables, and random effects used within the study

| **Analysis objective** | **Comparison** | **Response variable** | **Statistical method** | **Fixed effect / explanatory variable** | **Random effects / conditioning variables** |
| --- | --- | --- | --- | --- | --- |
| **Environmental differences between habitat types** | Rice paddies vs. wetlands | Conductivity, dissolved oxygen, water depth, pH, water temperature, water surface area | MANOVA (Pillai's Trace) | Habitat type (rice / wetland) | None |
| **Contribution of individual environmental variables** | Rice paddies vs. wetlands | Conductivity, dissolved oxygen, water depth, pH, water temperature, water surface area (analysed separately) | LMMs | Habitat type | Site; sampling period (year × sampling round) |
| **Environmental differences associated with ditch presence** | Paddies with ditches vs. paddies without ditches (centre-only & between paddy dataset) | Conductivity, dissolved oxygen, water depth, pH, water temperature | LMMs | Ditch presence (present/absent) | Site; sampling period |
| **Environmental differences within paddies** | Ditch vs. paddy centre (within-paddy dataset) | Conductivity, dissolved oxygen, water depth, pH, water temperature | LMMs | Sampling position (ditch/centre) | Site; sampling period |
| **Gamma diversity comparison** | Rice paddies vs. wetlands | Taxon richness | Rarefaction | Habitat type | None |
| **Alpha diversity comparison** | Rice paddies vs. wetlands | Taxon richness | GLMM, Poisson | Habitat type | Site; sampling period |
| **Alpha diversity comparison** | Rice paddies vs. wetlands | Density, Shannon diversity | LMMs | Habitat type | Site; sampling period |
| **Gamma diversity comparison** | Ditch presence and within-paddy comparisons | Taxon richness | Rarefaction | Ditch presence or sampling position | None |
| **Alpha diversity comparison** | Centre-only, between-paddy, and within-paddy datasets | Taxon richness | GLMM, Poisson | Ditch presence or sampling position | Site; sampling period |
| **Alpha diversity comparison** | Centre-only, between-paddy, and within-paddy datasets | Density, Shannon diversity | LMMs | Ditch presence or sampling position | Site; sampling period |
| **Taxon-habitat associations** | Rice paddies vs. wetlands | Taxon densities | Indicator species analysis (rpb) | Habitat type | Restricted permutations within site across sampling periods |
| **Community composition** | Rice paddies vs. wetlands | Macroinvertebrate community composition (Bray-Curtis dissimilarities) | Partial dbRDA | Habitat type, conductivity, dissolved oxygen, water depth, water surface area | Condition(site + year + sampling round); restricted permutations within site |
| **Taxon-ditch associations** | Centre-only, between-paddy, and within-paddy datasets | Taxon densities | Indicator species analysis (rpb) | Ditch presence or sampling position | Restricted permutations within site across sampling periods |

## Environmental factors

### Rice paddies vs. wetlands

Table S 3 The environmental variables measured in rice paddies and wetlands are presented with sample size (N), means, and standard deviation (SD). Results of the univariate normality with Shapiro-Wilk test (W-statistic) are also included. The p-values for each group and dependent variable combination are significant, indicating that the assumption of multivariate normality is not met.

| **Habitat** | **Variable** | unit | **N** | **Mean** | **SD** | **W- Statistic** | **P-value** |
| --- | --- | --- | --- | --- | --- | --- | --- |
| **Rice** | **Water surface area** | ha | 39 | 0.91 | 0.48 | 0.934 | **0.024** |
|  | **Conductivity** | µS/cm | 39 | 364.51 | 189.71 | 0.919 | **0.008** |
|  | **Water depth** | cm | 39 | 12.30 | 5.54 | 0.926 | **0.013** |
|  | **Dissolved oxygen** | mg/L | 39 | 8.39 | 3.69 | 0.850 | **<0.001** |
|  | **pH** |  | 39 | 7.87 | 0.68 | 0.888 | **<0.001** |
|  | **Water temperature** | °C | 39 | 24.58 | 3.57 | 0.986 | 0.905 |
| **Wetland** | **Water surface area** | ha | 40 | 0.26 | 0.51 | 0.560 | **<0.001** |
|  | **Conductivity** | µS/cm | 40 | 273.27 | 201.07 | 0.925 | **0.011** |
|  | **Water depth** | cm | 40 | 13.78 | 8.44 | 0.931 | **0.017** |
|  | **Dissolved oxygen** | mg/L | 40 | 7.63 | 3.69 | 0.953 | 0.100 |
|  | **pH** |  | 40 | 7.73 | 1.73 | 0.868 | **<0.001** |
|  | **Water temperature** | °C | 40 | 24.87 | 3.84 | 0.974 | 0.468 |

Table S 4 Multivariate normality assessed with Mardia’s test, in addition to the Shapiro-Wilk test. The skewness test showed significance (p < 0.05), indicating asymmetrical data distribution. The kurtosis test also indicated significant results, revealing heavy tails or outliers, therefore we tested further for outliers (Mahalanobis distance) and covariance homogeneity (Box’s M test).

| **Test** | **Statistic** | **p-value** | **Result** |
| --- | --- | --- | --- |
| **Skewness** | 202.18 | <0.001 | NO |
| **Kurtosis** | 4.67 | <0.001 | NO |
| **MV Normality** | NA | NA | NO |

Table S 5 Box’s M test

| Box’s M | **χ^2^** | Df | P-value |
| --- | --- | --- | --- |
|  | 65.768 | 21 | **<0.001** |

Table S 6 Results of Levene’s test. Because the results of Box’s M test were significant, meaning covariance is not homogeneous and the assumption violated, we tested the homogeneity of variance for each dependent variable individually with this test. Conductivity and pH have heterogeneous variance, as indicated by the significant p-values. The non-significant environmental variables follow the homogeneity of variance.

| **Variable** | **F-statistic** | **P-value** |
| --- | --- | --- |
| **Water surface area** | 1.18 | 0.281 |
| **Conductivity** | 14.75 | **<0.001** |
| **Water depth** | 0.75 | 0.389 |
| **Dissolved oxygen** | 0.04 | 0.847 |
| **pH** | 8.68 | **0.004** |
| **Water temperature** | 0.94 | 0.335 |

Table S 7 MANOVA Univariate ANOVA result table of the environmental parameters, with 1 excluded Outlier, detected with Mahalanobis distances check. Habitat categories were rice paddies and wetlands.

| **Variable** |  | **Df** | **Sum Sq** | **F-value** | **P-value** |
| --- | --- | --- | --- | --- | --- |
| **Water surface area** | Habitat | 1 | 3.85 | 51.58 | **<0.001** |
|  | Residuals | 77 | 5.75 |  |  |
| **Conductivity** | Habitat | 1 | 3.32 | 12.48 | **0.001** |
|  | Residuals | 77 | 20.48 |  |  |
| **Water depth** | Habitat | 1 | 0.02 | 0.05 | 0.830 |
|  | Residuals | 77 | 26.16 |  |  |
| **Dissolved oxygen** | Habitat | 1 | 0.10 | 0.40 | 0.529 |
|  | Residuals | 77 | 19.67 |  |  |
| **pH** | Habitat | 1 | 0.38 | 0.22 | 0.642 |
|  | Residuals | 77 | 134.42 |  |  |
| **Water temperature** | Habitat | 1 | 0.00 | 0.09 | 0.770 |
|  | Residuals | 77 | 1.61 |  |  |

Table S 8 Comparison of environmental variables between rice paddies and wetlands using linear mixed-effects models (LMMs). Habitat type (rice paddy vs. wetland) was included as a fixed effect. Random effects accounting for repeated sampling (Site ID and Sampling Campaign) were included for all variables except water surface area, for which only Site ID was used because area did not vary over time. To ensure comparability with the MANOVA results, the same outlier identified by the Mahalanobis distance test was excluded from all LMMs. Marg. R² = marginal R²; Cond. R² = conditional R²

Table S 9 Comparison of environmental variables between rice paddies and wetlands based on linear mixed-effects models (LMMs). Habitat type was included as a fixed effect, with Site ID and Sampling Campaign as random effects (except for water surface area, which included Site ID only). (F-) Statistic and p-values refer to the effect of habitat type.

Figure S 1 Correlation of environmental variables in rice paddies (red) and wetlands (blue). Upper panel shows Pearson’s correlation with significance level (asterisks) for overall (grey) and per habitat. Lower plots show pairwise scatterplots of environmental variables per habitat. In the middle are density plots per habitat. All variables but pH were log+1 transformed prior.

Figure S 2 Environmental variables for rice paddies and wetlands over time (e.g., year and sampling round, such as 1 - July or 2 - August).

### Rice paddies & ditches

Figure S 3 Summary of environmental measurements in paddy centers and ditches (side) in time.

Table S 10 Differences in environmental variables among rice paddies with and without ditches based on linear mixed-effects models (LMMs). Ditch presence (present vs. absent) was included as a fixed effect. P-values for the fixed effects were adjusted using the false discovery rate (Benjamini–Hochberg) method.

| ***Dataset*** | ***Predictor*** | ***Variable*** | ***Sum Sq*** | ***Num Df*** | ***Den Df*** | ***F*** | ***BH p-value*** |
| --- | --- | --- | --- | --- | --- | --- | --- |
| Center only | Ditch presence | Conductivity | 0.037 | 1 | 9.51 | 1.06 | 0.547 |
|  |  | Water depth | 0.983 | 1 | 7.68 | 4.25 | 0.373 |
|  |  | Dissolved oxygen | 0.300 | 1 | 7.91 | 2.21 | 0.439 |
|  |  | pH | 0.171 | 1 | 35.22 | 0.25 | 0.623 |
|  |  | Water temperature | 0.005 | 1 | 8.91 | 0.50 | 0.623 |
| Merged Center + Ditch | Ditch presence | Conductivity | 0.018 | 1 | 9.62 | 0.29 | 0.806 |
|  |  | Water depth | 0.120 | 1 | 8.21 | 0.62 | 0.806 |
|  |  | Dissolved oxygen | 0.078 | 1 | 8.93 | 0.56 | 0.806 |
|  |  | pH | 0.029 | 1 | 36.34 | 0.06 | 0.806 |
|  |  | Water temperature | 0.002 | 1 | 9.34 | 0.21 | 0.806 |
| only Ditch present | Position in Paddy | Conductivity | 0.040 | 1 | 37.93 | 0.49 | 0.595 |
|  |  | Water depth | 5.880 | 1 | 39.53 | 19.71 | **<0.001** |
|  |  | Dissolved oxygen | 0.853 | 1 | 38.99 | 5.19 | **0.071** |
|  |  | pH | 0.253 | 1 | 43.00 | 0.29 | 0.595 |
|  |  | Water temperature | 0.035 | 1 | 37.94 | 2.33 | 0.225 |

## Macroinvertebrate diversity

### Rice paddies & ditches

Figure S 4 Rarefaction curves for the comparison between rice paddies with and without ditches, and within paddies with a ditch present. Results of the comparison between paddies are shown in A and B; results of the Centre only dataset are shown in C and D; and results of the comparison within paddies with ditches are shown in E and F. Abundance-based rarefaction is shown in A, C and E, and sample-size-based rarefaction in B, D and F.

## Community composition

### Rice paddies vs. wetlands

Table S 11 Macroinvertebrates counts in 11 rice paddies and 11 wetlands over the years 2022-23.

|  | **Rice paddy** | | | | | |  | **Wetland** | | | | | |
| --- | --- | --- | --- | --- | --- | --- | --- | --- | --- | --- | --- | --- | --- |
| **Taxa** | **2022** | |  | **2023** | | **Total** |  | **2022** | |  | **2023** | | **Total** |
|  | **Jul** | **Aug** |  | **Jul** | **Aug** |  |  | **Jul** | **Aug** |  | **Jul** | **Aug** |  |
| ***Sympetrum striolatum*** | 0 | 0 |  | 0 | 0 | **0** |  | 0 | 0 |  | 6 | 0 | **6** |
| ***Sympetrum fonscolombii*** | 141 | 84 |  | 171 | 19 | **415** |  | 23 | 35 |  | 3 | 11 | **72** |
| ***Sympetrum depressiusculum*** | 0 | 0 |  | 0 | 0 | **0** |  | 1 | 0 |  | 2 | 1 | **4** |
| ***Sympetrum sp*** | 0 | 0 |  | 0 | 9 | **9** |  | 2 | 0 |  | 1 | 7 | **10** |
| ***Crocothemis erythraea*** | 22 | 84 |  | 2 | 27 | **135** |  | 7 | 54 |  | 0 | 8 | **69** |
| ***Orthetrum cancellatum*** | 116 | 111 |  | 105 | 89 | **421** |  | 1 | 8 |  | 1 | 5 | **15** |
| ***Orthetrum brunneum*** | 62 | 49 |  | 42 | 30 | **183** |  | 2 | 7 |  | 0 | 7 | **16** |
| ***Orthetrum albistylum*** | 47 | 44 |  | 25 | 12 | **128** |  | 24 | 10 |  | 6 | 13 | **53** |
| ***Orthetrum sp*** | 34 | 9 |  | 48 | 70 | **161** |  | 3 | 17 |  | 5 | 10 | **35** |
| ***Libellula quadrimaculata*** | 4 | 1 |  | 1 | 13 | **19** |  | 1 | 2 |  | 1 | 3 | **7** |
| ***Libellula depressa*** | 110 | 140 |  | 123 | 64 | **437** |  | 17 | 19 |  | 17 | 4 | **57** |
| ***Libellulinae*** | 0 | 0 |  | 61 | 0 | **61** |  | 0 | 0 |  | 1 | 18 | **19** |
| ***Anax parthenope*** | 6 | 14 |  | 3 | 15 | **38** |  | 1 | 0 |  | 0 | 1 | **2** |
| ***Anax imperator*** | 18 | 29 |  | 8 | 18 | **73** |  | 11 | 4 |  | 5 | 2 | **22** |
| ***Anax epiphiger*** | 1 | 5 |  | 0 | 0 | **6** |  | 0 | 0 |  | 0 | 0 | **0** |
| ***Anax sp*** | 37 | 27 |  | 44 | 60 | **168** |  | 19 | 6 |  | 2 | 4 | **31** |
| ***Aeshna sp*** | 1 | 0 |  | 0 | 1 | **2** |  | 1 | 1 |  | 4 | 0 | **6** |
| ***Ischnura sp*** | 32 | 2 |  | 60 | 3 | **97** |  | 11 | 0 |  | 7 | 1 | **19** |
| ***Enallagma cyathigerum*** | 0 | 15 |  | 24 | 46 | **85** |  | 0 | 0 |  | 1 | 11 | **12** |
| ***Coenagrion sp*** | 1 | 91 |  | 18 | 21 | **131** |  | 31 | 73 |  | 5 | 29 | **138** |
| ***Coenangrionidae*** | 11 | 71 |  | 32 | 27 | **141** |  | 35 | 45 |  | 10 | 34 | **124** |
| ***Anisus*** | 1 | 0 |  | 1 | 0 | **2** |  | 1 | 0 |  | 0 | 0 | **1** |
| ***Bithynia*** | 0 | 0 |  | 0 | 0 | **0** |  | 12 | 8 |  | 0 | 0 | **20** |
| ***Gyraulus*** | 25 | 119 |  | 85 | 43 | **272** |  | 11 | 64 |  | 26 | 185 | **286** |
| ***Stagnicola*** | 0 | 0 |  | 0 | 0 | **0** |  | 0 | 0 |  | 4 | 0 | **4** |
| ***Physa*** | 993 | 1102 |  | 3510 | 3756 | **9361** |  | 21 | 89 |  | 32 | 30 | **172** |
| ***Planorbis*** | 0 | 0 |  | 0 | 4 | **4** |  | 5 | 40 |  | 2 | 11 | **58** |
| ***Radix*** | 0 | 0 |  | 0 | 13 | **13** |  | 40 | 24 |  | 62 | 22 | **148** |
| ***Lithoglyphus naticoides*** | 0 | 0 |  | 0 | 0 | **0** |  | 0 | 0 |  | 5 | 0 | **5** |
| ***Pisidium*** | 0 | 0 |  | 0 | 0 | **0** |  | 0 | 17 |  | 21 | 16 | **54** |
| ***Sphaerium*** | 0 | 0 |  | 0 | 0 | **0** |  | 4 | 3 |  | 0 | 24 | **31** |
| ***Gammarus*** | 0 | 4 |  | 13 | 0 | **17** |  | 41 | 30 |  | 27 | 81 | **179** |
| ***Asellus aquaticus*** | 0 | 0 |  | 0 | 0 | **0** |  | 151 | 323 |  | 37 | 165 | **676** |
| ***Cloeon*** | 2191 | 1550 |  | 2809 | 1878 | **8428** |  | 743 | 341 |  | 261 | 1944 | **3289** |
| ***Caenis*** | 1 | 0 |  | 12 | 0 | **13** |  | 23 | 8 |  | 19 | 0 | **50** |
| ***other Adephaga*** | 3 | 2 |  | 8 | 10 | **23** |  | 12 | 27 |  | 10 | 21 | **70** |
| ***Laccophilus*** | 92 | 33 |  | 122 | 25 | **272** |  | 28 | 30 |  | 17 | 15 | **90** |
| ***Hydroporinae*** | 23 | 3 |  | 29 | 9 | **64** |  | 22 | 48 |  | 24 | 15 | **109** |
| ***other dytiscidae*** | 8 | 2 |  | 1 | 11 | **22** |  | 9 | 4 |  | 1 | 0 | **14** |
| ***other Polyphaga*** | 0 | 2 |  | 0 | 0 | **2** |  | 57 | 32 |  | 2 | 2 | **93** |
| ***Hydrophilidae*** | 25 | 6 |  | 24 | 9 | **64** |  | 21 | 20 |  | 18 | 0 | **59** |
| ***Tipuloidea*** | 1 | 0 |  | 4 | 0 | **5** |  | 0 | 0 |  | 0 | 0 | **0** |
| ***Psychodidae*** | 0 | 0 |  | 4 | 0 | **4** |  | 0 | 0 |  | 0 | 1 | **1** |
| ***Chaoboridae*** | 8 | 1 |  | 2 | 27 | **38** |  | 132 | 13 |  | 6 | 8 | **159** |
| ***Dixidae*** | 0 | 1 |  | 0 | 0 | **1** |  | 0 | 0 |  | 0 | 5 | **5** |
| ***Anophelinae*** | 43 | 58 |  | 60 | 58 | **219** |  | 1 | 18 |  | 2 | 34 | **55** |
| ***Culicinae*** | 48 | 4 |  | 119 | 158 | **329** |  | 0 | 16 |  | 0 | 56 | **72** |
| ***Ceratopogonidae*** | 2 | 2 |  | 36 | 49 | **89** |  | 0 | 0 |  | 11 | 201 | **212** |
| ***Orthocladiinae*** | 16 | 2 |  | 0 | 170 | **188** |  | 4 | 2 |  | 6 | 0 | **12** |
| ***Tanypodinae*** | 543 | 336 |  | 740 | 608 | **2227** |  | 35 | 95 |  | 61 | 291 | **482** |
| ***Chironomini*** | 127 | 110 |  | 197 | 379 | **813** |  | 10 | 105 |  | 22 | 48 | **185** |
| ***Tanytarsini*** | 2 | 4 |  | 179 | 159 | **344** |  | 0 | 5 |  | 6 | 19 | **30** |
| ***Brachyceres*** | 0 | 3 |  | 10 | 1 | **14** |  | 0 | 2 |  | 7 | 6 | **15** |
| ***Gerromorpha*** | 0 | 3 |  | 10 | 1 | **14** |  | 0 | 2 |  | 7 | 6 | **15** |
| ***Corixidae*** | 705 | 235 |  | 803 | 52 | **1795** |  | 39 | 225 |  | 14 | 10 | **288** |
| ***Micronecta*** | 0 | 0 |  | 0 | 39 | **39** |  | 10 | 45 |  | 95 | 480 | **630** |
| ***Notonectoidea*** | 0 | 0 |  | 0 | 3 | **3** |  | 11 | 3 |  | 13 | 10 | **37** |
| ***Plea minutissima*** | 1 | 1 |  | 7 | 4 | **13** |  | 124 | 151 |  | 62 | 21 | **358** |
| ***Trichoptera*** | 0 | 0 |  | 1 | 0 | **1** |  | 1 | 2 |  | 6 | 2 | **11** |
| ***Sialis*** | 0 | 0 |  | 0 | 0 | **0** |  | 1 | 1 |  | 1 | 0 | **3** |

Table S 12 Taxon association strength of macroinvertebrates to the habitat types, rice paddy and wetland. Given are the point biserial correlation coefficient (rpb) and FDR corrected p-values.

| **Habitat** | **Group** | **Taxon** | **r_pb_** | **P-fdr** |
| --- | --- | --- | --- | --- |
| **Rice** | **Gastropoda** | *Anisus* | 0.02 | 0.791 |
|  |  | ***Physa*** | 0.35 | **0.004** |
|  | **Odonata** | *Enallagma cyathigerum* | 0.04 | 0.698 |
|  |  | *Libellula quadrimaculata* | 0.06 | 0.703 |
|  |  | *Ischnura sp* | 0.08 | 0.698 |
|  |  | *Anax sp* | 0.10 | 0.688 |
|  |  | *Anax epiphiger* | 0.12 | 0.641 |
|  |  | *Libellula depressa* | 0.19 | 0.184 |
|  |  | *Orthetrum brunneum* | 0.22 | 0.338 |
|  |  | *Anax parthenope* | 0.29 | 0.055 |
|  |  | ***Orthetrum cancellatum*** | 0.42 | **0.004** |
|  | **Diptera** | *Psychodidae* | 0.00 | 0.845 |
|  |  | *Culicinae* | 0.02 | 0.791 |
|  |  | *Tanypodinae* | 0.10 | 0.641 |
|  |  | *Orthocladiinae* | 0.11 | 0.339 |
|  |  | *Tipuloidea* | 0.16 | 0.451 |
|  |  | *Tanytarsini* | 0.16 | 0.424 |
|  | **Hemiptera** | *Corixidae* | 0.16 | 0.350 |
| **Wetland** | **Gastropoda** | *Radix* | -0.20 | 0.641 |
|  |  | *Planorbis* | -0.16 | 0.055 |
|  |  | *Bithynia* | -0.16 | 0.641 |
|  |  | *Lithoglyphus naticoides* | -0.11 | 0.791 |
|  |  | *Stagnicola* | -0.11 | 0.791 |
|  |  | *Gyraulus* | -0.05 | 0.791 |
|  | **Bivalvia** | *Pisidium* | -0.23 | 0.184 |
|  |  | ***Sphaerium*** | -0.23 | **0.042** |
|  | **Amphipod** | *Gammarus* | -0.20 | 0.103 |
|  | **Isopoda** | ***Asellus aquaticus*** | -0.20 | **0.048** |
|  | **Ephemeroptera** | ***Caenis*** | -0.29 | **0.045** |
|  |  | *Cloeon* | 0.07 | 0.791 |
|  | **Odonata** | *Coenangrionidae* | -0.21 | 0.171 |
|  |  | *Sympetrum depressiusculum* | -0.19 | 0.698 |
|  |  | *Sympetrum striolatum* | -0.16 | 0.698 |
|  |  | *Orthetrum albistylum* | -0.16 | 0.350 |
|  |  | *Coenagrion sp* | -0.16 | 0.362 |
|  |  | *Sympetrum sp* | -0.14 | 0.641 |
|  |  | *Aeshna sp* | -0.13 | 0.544 |
|  |  | *Crocothemis erythraea* | -0.10 | 0.698 |
|  |  | *Libellulinae* | -0.07 | 0.791 |
|  |  | *Anax imperator* | -0.05 | 0.698 |
|  |  | *Sympetrum fonscolombii* | -0.01 | 0.791 |
|  |  | *Orthetrum sp* | 0.02 | 0.698 |
|  | **Megaloptera** | *Sialis* | -0.11 | 0.641 |
|  | **Trichoptera** | *Trichoptera* | -0.19 | 0.339 |
|  | **Coleoptera** | *other Adephaga* | -0.28 | 0.055 |
|  |  | *Hydroporinae* | -0.19 | 0.140 |
|  |  | *other Polyphaga* | -0.19 | 0.101 |
|  |  | *Hydrophilidae* | -0.18 | 0.339 |
|  |  | *other dytiscidae* | -0.04 | 0.791 |
|  |  | *Laccophilus* | 0.06 | 0.791 |
|  | **Diptera** | *Brachyceres* | -0.23 | 0.184 |
|  |  | *Chaoboridae* | -0.12 | 0.641 |
|  |  | *Dixidae* | -0.11 | 0.845 |
|  |  | *Ceratopogonidae* | -0.09 | 0.698 |
|  |  | *Anophelinae* | 0.00 | 0.791 |
|  |  | *Chironomini* | 0.05 | 0.791 |
|  | **Hemiptera** | ***Notonectoidea*** | -0.34 | **0.005** |
|  |  | ***Plea minutissima*** | -0.33 | **0.004** |
|  |  | *Gerromorpha* | -0.23 | 0.184 |
|  |  | ***Micronecta*** | -0.14 | **0.036** |

Table S 13 Result of permutation test of dispersion with betadisper() and below the mean & SD per habitat.

|  | **Df** | **Sumo f Sq** | **F-value** | **No. Perm.** | **p-value** |
| --- | --- | --- | --- | --- | --- |
| Habitat type | 1 | 0.186 | 78.71 | **999** | 0.001 |
| Residuals | 77 | 0.182 |  |  |  |
|  |  | **Mean** | **SD** |  |  |
| **Rice paddy** |  | 0.51 | 0.050 |  |  |
| **Wetland** |  | 0.61 | 0.046 |  |  |

Table S 14 Results of forward selection of explanatory variables with adjusted R² values for multiple testing. The initial full model included macroinvertebrate dissimilarity as a function of habitat, surface area, conductivity, pH, dissolved oxygen, temperature, water depth, and partialled out condition (site ID, year, sampling month). A stopping criterion of R² = 0.101 equal to the full model was applied. The final model retained habitat, surface area, conductivity, dissolved oxygen, water depth, and excluding pH and temperature.

|  | **Adjusted R²** | **Df** | **AIC** | **F-value** | **P-value** |
| --- | --- | --- | --- | --- | --- |
| **Habitat type** | 0.047 | 1 | 257.86 | 4.70 | **0.005** |
| **Water surface area** | 0.069 | 1 | 256.61 | 2.78 | **0.005** |
| **Conductivity** | 0.088 | 1 | 255.56 | 2.56 | **0.005** |
| **Dissolved oxygen** | 0.095 | 1 | 255.63 | 1.58 | **0.036** |
| **Water depth** | 0.100 | 1 | 255.90 | 1.40 | 0.070 |

Table S 15 Eigenvalues and the proportion of constrained and total variation explained by each dbRDA axis for the comparison between rice paddies and wetlands. The last column shows the cumulative percentage of explained variation for the constrained axis.

| **Axis** | **Eigenvalue** | **% Constrained** | **% Total** | **Cumulative % Constrained** |
| --- | --- | --- | --- | --- |
| ***dbRDA1*** | 1.44 | 39.1 | 6.9 | 39.1 |
| ***dbRDA2*** | 0.86 | 23.2 | 4.1 | 62.3 |
| ***dbRDA3*** | 0.73 | 19.8 | 3.5 | 82.1 |
| ***dbRDA4*** | 0.35 | 9.6 | 1.7 | 91.6 |
| ***dbRDA5*** | 0.31 | 8.4 | 1.5 | 100.0 |

### Rice paddies & ditches

Figure S 5 Association strength (r_pb_) of macroinvertebrates overall in rice paddies (Merged dataset) with and without ditch present Confidence intervals by bootstrap (n = 1000). P-values were initially computed using permutation methods and corrected with the default Sidak method, accounting for two group comparisons per species. To control for the total number of taxa tested we adjusted the p values with Benjamini-Hochberg method for false discovery rate, but no taxa were significant.

Figure S 6 Association strength (r_pb_) of macroinvertebrates in paddy centers (Center only dataset) Confidence intervals by bootstrap (n = 1000). Significance level tested with permutation (n=9999) restricted to permute within site ID. P-values were initially computed using permutation methods and corrected with the default Sidak method, accounting for two group comparisons per species. To control for the total number of taxa tested, we adjusted p-values with Benjamini–Hochberg method for FDR control.

Table S 16 Association strength for Ditch paddies only dataset

| **Within paddies with Ditch** | | | | |
| --- | --- | --- | --- | --- |
| **Position in Paddy** | **Group** | **Taxa** | **rpb** | **P-fdr** |
| **Ditch** | **Gastropoda** | *Anisus* | 0.00 | 0.940 |
|  |  | *Physa* | -0.08 | 0.836 |
|  |  | *Radix* | -0.19 | 0.791 |
|  |  | *Gyraulus* | -0.20 | 0.720 |
|  | **Bivalvia** | *Pisidium* | -0.15 | 0.836 |
|  |  | *Sphaerium* | -0.15 | 0.836 |
|  | **Amphipod** | *Gammarus* | -0.02 | 0.836 |
|  | **Isopoda** | *Asellus aquaticus* | -0.15 | 0.836 |
|  | **Ephemeroptera** | *Cloeon* | -0.19 | 0.791 |
|  | **Odonata** | *Coenangrionidae* | -0.07 | 0.836 |
|  |  | *Aeshna sp* | -0.07 | 0.836 |
|  |  | *Anax sp* | -0.10 | 0.791 |
|  |  | *Ischnura sp* | -0.13 | 0.791 |
|  |  | *Libellula quadrimaculata* | -0.15 | 0.836 |
|  |  | *Enallagma cyathigerum* | -0.20 | 0.720 |
|  |  | *Libellula depressa* | -0.20 | 0.653 |
|  |  | *Anax imperator* | -0.29 | 0.532 |
|  | **Megaloptera** | *Sialis* | -0.15 | 0.870 |
|  | **Trichoptera** | *Trichoptera* | -0.21 | 0.836 |
|  | **Coleoptera** | *other Polyphaga* | 0.00 | 0.935 |
|  |  | *Hydroporinae* | -0.05 | 0.836 |
|  |  | *Hydrophilidae* | -0.08 | 0.836 |
|  |  | *Laccophilus* | -0.15 | 0.836 |
|  |  | *other Adephaga* | -0.26 | 0.720 |
|  | **Diptera** | *Dixidae* | 0.00 | 0.935 |
|  |  | *Ceratopogonidae* | -0.12 | 0.836 |
|  |  | *Chaoboridae* | -0.17 | 0.791 |
|  | **Hemiptera** | *Corixidae* | -0.12 | 0.791 |
|  |  | *Plea minutissima* | -0.23 | 0.720 |
|  |  | *Notonectoidea* | -0.35 | 0.208 |
| **Paddy centre** | **Odonata** | ***Orthetrum brunneum*** | 0.39 | **0.010** |
|  |  | *Anax parthenope* | 0.30 | 0.532 |
|  |  | *Orthetrum sp* | 0.29 | 0.532 |
|  |  | *Crocothemis erythraea* | 0.18 | 0.791 |
|  |  | *Orthetrum albistylum* | 0.17 | 0.791 |
|  |  | *Sympetrum sp* | 0.15 | 0.870 |
|  |  | *Coenagrion sp* | 0.14 | 0.836 |
|  |  | *Libellulinae* | 0.13 | 0.836 |
|  |  | *Sympetrum fonscolombii* | 0.13 | 0.836 |
|  |  | *Orthetrum cancellatum* | 0.13 | 0.791 |
|  | **Coleoptera** | *other dytiscidae* | 0.16 | 0.836 |
|  | **Diptera** | *Culicinae* | 0.18 | 0.791 |
|  |  | *Anophelinae* | 0.17 | 0.791 |
|  |  | *Tanytarsini* | 0.17 | 0.791 |
|  |  | *Orthocladiinae* | 0.14 | 0.836 |
|  |  | *Psychodidae* | 0.11 | 0.836 |
|  |  | *Brachyceres* | 0.09 | 0.836 |
|  |  | *Tanypodinae* | 0.08 | 0.836 |
|  |  | *Tipuloidea* | 0.07 | 0.836 |
|  |  | *Chironomini* | 0.01 | 0.836 |
|  | **Hemiptera** | *Micronecta* | 0.15 | 0.836 |
|  |  | *Gerromorpha* | 0.09 | 0.836 |

Table S 17 Association strength for Centre only dataset

| **Center-only dataset** | | | | |
| --- | --- | --- | --- | --- |
| **Ditch presence in paddy** | **Group** | **Taxa** | **rpb** | **P-fdr** |
| **Absent** | **Gastropoda** | *Gyraulus* | 0.26 | 1.000 |
|  |  | *Planorbis* | 0.19 | 1.000 |
|  | **Ephemeroptera** | *Caenis* | 0.27 | 1.000 |
|  |  | *Cloeon* | 0.06 | 1.000 |
|  | **Odonata** | *Anax imperator* | 0.36 | 1.000 |
|  |  | *Anax epiphiger* | 0.19 | 1.000 |
|  |  | *Sympetrum fonscolombii* | 0.11 | 1.000 |
|  |  | *Libellula depressa* | 0.11 | 1.000 |
|  |  | *Libellulinae* | 0.09 | 1.000 |
|  |  | *Orthetrum cancellatum* | 0.09 | 1.000 |
|  |  | *Ischnura sp* | 0.05 | 1.000 |
|  | **Trichoptera** | *Trichoptera* | 0.19 | 1.000 |
|  | **Coleoptera** | *other Adephaga* | 0.28 | 1.000 |
|  |  | *Hydroporinae* | 0.06 | 1.000 |
|  |  | *other dytiscidae* | 0.06 | 1.000 |
|  |  | *Laccophilus* | 0.05 | 1.000 |
|  |  | *Hydrophilidae* | 0.02 | 1.000 |
|  |  | *other Polyphaga* | 0.00 | 1.000 |
|  | **Diptera** | *Chaoboridae* | 0.07 | 1.000 |
|  |  | *Tanypodinae* | 0.05 | 1.000 |
|  |  | *Chironomini* | 0.01 | 1.000 |
|  | **Hemiptera** | *Notonectoidea* | 0.15 | 1.000 |
|  |  | *Corixidae* | 0.10 | 1.000 |
| **Present** | **Gastropoda** | *Physa* | -0.03 | 1.000 |
|  |  | *Anisus* | -0.10 | 1.000 |
|  |  | *Radix* | -0.17 | 1.000 |
|  | **Amphipod** | *Gammarus* | -0.16 | 1.000 |
|  | **Odonata** | *Anax sp* | -0.04 | 1.000 |
|  |  | *Crocothemis erythraea* | -0.06 | 1.000 |
|  |  | *Anax parthenope* | -0.11 | 1.000 |
|  |  | *Aeshna sp* | -0.15 | 1.000 |
|  |  | *Sympetrum sp* | -0.15 | 1.000 |
|  |  | *Orthetrum albistylum* | -0.17 | 1.000 |
|  |  | *Coenangrionidae* | -0.17 | 1.000 |
|  |  | *Coenagrion sp* | -0.20 | 1.000 |
|  |  | *Orthetrum sp* | -0.21 | 1.000 |
|  |  | *Orthetrum brunneum* | -0.22 | 1.000 |
|  |  | *Libellula quadrimaculata* | -0.22 | 1.000 |
|  |  | *Enallagma cyathigerum* | -0.25 | 1.000 |
|  | **Diptera** | *Culicinae* | -0.06 | 1.000 |
|  |  | *Tanytarsini* | -0.09 | 1.000 |
|  |  | *Orthocladiinae* | -0.14 | 1.000 |
|  |  | *Tipuloidea* | -0.14 | 1.000 |
|  |  | *Dixidae* | -0.15 | 1.000 |
|  |  | *Psychodidae* | -0.15 | 1.000 |
|  |  | *Ceratopogonidae* | -0.20 | 1.000 |
|  |  | *Brachyceres* | -0.22 | 1.000 |
|  |  | *Anophelinae* | -0.25 | 1.000 |
|  | **Hemiptera** | *Plea minutissima* | -0.01 | 1.000 |
|  |  | *Micronecta* | -0.15 | 1.000 |
|  |  | *Gerromorpha* | -0.22 | 1.000 |

Table S 18 Association strength for the between paddies (Centers+ Ditches) dataset

| **Between paddy (Center + Ditch)** | | | | |
| --- | --- | --- | --- | --- |
| **Ditch presence in paddy** | **Group** | **Taxa** | **rpb** | **P-value** |
| **Absent** | **Gastropoda** | *Planorbis* | 0.19 | 1.000 |
|  |  | *Gyraulus* | 0.11 | 1.000 |
|  | **Ephemeroptera** | *Caenis* | 0.27 | 1.000 |
|  | **Odonata** | *Anax imperator* | 0.21 | 1.000 |
|  |  | *Anax epiphiger* | 0.19 | 1.000 |
|  |  | *Sympetrum fonscolombii* | 0.19 | 1.000 |
|  |  | *Orthetrum cancellatum* | 0.15 | 1.000 |
|  |  | *Libellulinae* | 0.14 | 1.000 |
|  |  | *Crocothemis erythraea* | 0.05 | 1.000 |
|  |  | *Anax parthenope* | 0.04 | 1.000 |
|  |  | *Libellula depressa* | 0.03 | 1.000 |
|  | **Coleoptera** | *other dytiscidae* | 0.10 | 1.000 |
|  |  | *other Adephaga* | 0.06 | 1.000 |
|  |  | *other Polyphaga* | 0.01 | 1.000 |
|  |  | *Hydroporinae* | 0.01 | 1.000 |
|  | **Diptera** | *Tanypodinae* | 0.09 | 1.000 |
|  |  | *Culicinae* | 0.05 | 1.000 |
|  |  | *Chironomini* | 0.02 | 1.000 |
|  | **Hemiptera** | *Corixidae* | 0.03 | 1.000 |
| **Present** | **Gastropoda** | *Physa* | -0.09 | 1.000 |
|  |  | *Anisus* | -0.13 | 1.000 |
|  |  | *Radix* | -0.23 | 1.000 |
|  | **Bivalvia** | *Pisidium* | -0.15 | 1.000 |
|  |  | *Sphaerium* | -0.15 | 1.000 |
|  | **Amphipod** | *Gammarus* | -0.19 | 1.000 |
|  | **Isopoda** | *Asellus aquaticus* | -0.15 | 1.000 |
|  | **Ephemeroptera** | *Cloeon* | -0.12 | 1.000 |
|  | **Odonata** | *Ischnura sp* | -0.03 | 1.000 |
|  |  | *Orthetrum brunneum* | -0.05 | 1.000 |
|  |  | *Orthetrum albistylum* | -0.07 | 1.000 |
|  |  | *Anax sp* | -0.08 | 1.000 |
|  |  | *Orthetrum sp* | -0.11 | 1.000 |
|  |  | *Sympetrum sp* | -0.15 | 1.000 |
|  |  | *Aeshna sp* | -0.20 | 1.000 |
|  |  | *Coenagrion sp* | -0.20 | 1.000 |
|  |  | *Libellula quadrimaculata* | -0.22 | 1.000 |
|  |  | *Enallagma cyathigerum* | -0.23 | 1.000 |
|  |  | *Coenangrionidae* | -0.26 | 1.000 |
|  | **Megaloptera** | *Sialis* | -0.15 | 1.000 |
|  | **Trichoptera** | *Trichoptera* | -0.18 | 1.000 |
|  | **Coleoptera** | *Hydrophilidae* | -0.06 | 1.000 |
|  |  | *Laccophilus* | -0.09 | 1.000 |
|  | **Diptera** | *Tanytarsini* | 0.00 | 1.000 |
|  |  | *Chaoboridae* | -0.12 | 1.000 |
|  |  | *Orthocladiinae* | -0.14 | 1.000 |
|  |  | *Dixidae* | -0.15 | 1.000 |
|  |  | *Psychodidae* | -0.15 | 1.000 |
|  |  | *Ceratopogonidae* | -0.16 | 1.000 |
|  |  | *Tipuloidea* | -0.18 | 1.000 |
|  |  | *Anophelinae* | -0.19 | 1.000 |
|  |  | *Brachyceres* | -0.29 | 1.000 |
|  | **Hemiptera** | *Micronecta* | -0.15 | 1.000 |
|  |  | *Notonectoidea* | -0.19 | 1.000 |
|  |  | *Plea minutissima* | -0.24 | 1.000 |
|  |  | *Gerromorpha* | -0.29 | 1.000 |

Figure S 7 Correlation of habitat association strength (r_pb_) for macroinvertebrate taxa between wetlands and rice paddies (x-axis) and within rice paddies between ditch and paddy centre (y-axis). Each point represents one taxon’s association strength. Dashed vertical and horizontal lines indicate r_pb_ = 0, separating positive associations with rice paddies or paddy centres from negative associations with wetland and ditch communities. A significant positive Pearson correlation was detected between the two compared datasets (r = 0.35, p = 0.011, conf n = 52 taxa), indicating consistent habitat associations of taxa across rice paddies and wetland habitats. Taxa are labelled using abbreviations derived from the first two letters of each word for multi-word taxa and the first four letters for single-word taxa (Taxa list provided in Tab S15-17, and for Hydr1 = Hydrophilidae; Hydr2 = Hydroporinae; Tany1 = Tanypodinae; Tany2 = Tanytarsini). The lower, left blue quadrant displays the taxa associated to Ditch and Wetland areas.
